# Microglia-Derived Cytokines/Chemokines Are Involved in the Enhancement of LPS-Induced Loss of Nigrostriatal Dopaminergic Neurons in DJ-1 Knockout Mice

**DOI:** 10.1371/journal.pone.0151569

**Published:** 2016-03-16

**Authors:** Chia-Hung Chien, Ming-Jen Lee, Houng-Chi Liou, Horng-Huei Liou, Wen-Mei Fu

**Affiliations:** 1 Department of Life Science, College of Life Science, National Taiwan University, Taipei, Taiwan; 2 Department of Neurology, National Taiwan University Hospital, Taipei, Taiwan; 3 Pharmacological Institute, College of Medicine, National Taiwan University, Taipei, Taiwan; 4 Drug Research Center, College of Medicine, National Taiwan University, Taipei, Taiwan; Indiana School of Medicine, UNITED STATES

## Abstract

Mutation of DJ-1 (PARK7) has been linked to the development of early-onset Parkinson’s disease (PD). However, the underlying molecular mechanism is still unclear. This study is aimed to compare the sensitivity of nigrostriatal dopaminergic neurons to lipopolysaccharide (LPS) challenge between DJ-1 knockout (KO) and wild-type (WT) mice, and explore the underlying cellular and molecular mechanisms. Our results found that the basal levels of interferon (IFN)-γ (the hub cytokine) and interferon-inducible T-cell alpha chemoattractant (I-TAC) (a downstream mediator) were elevated in the substantia nigra of DJ-1 KO mice and in microglia cells with DJ-1 deficiency, and the release of cytokine/chemokine was greatly enhanced following LPS administration in the DJ-1 deficient conditions. In addition, direct intranigral LPS challenge caused a greater loss of nigrostriatal dopaminergic neurons and striatal dopamine content in DJ-1 KO mice than in WT mice. Furthermore, the sensitization of microglia cells to LPS challenge to release IFN-γ and I-TAC was via the enhancement of NF-κB signaling, which was antagonized by NF-κB inhibitors. LPS-induced increase in neuronal death in the neuron-glia co-culture was enhanced by DJ-1 deficiency in microglia, which was antagonized by the neutralizing antibodies against IFN-γ or I-TAC. These results indicate that DJ-1 deficiency sensitizes microglia cells to release IFN-γ and I-TAC and causes inflammatory damage to dopaminergic neurons. The interaction between the genetic defect (i.e. DJ-1) and inflammatory factors (e.g. LPS) may contribute to the development of PD.

## Introduction

Parkinson’s disease (PD) is characterized by the loss of dopaminergic neurons in the substantia nigra pars compacta, leading to the inhibition of nigrostriatal neural projection and appearance of motor symptoms, such as muscular weakness, rigidity, tremor, and bradykinesia [1, 2]. The neuronal loss in PD is known to be caused by a complex interaction of genetic and environmental factors, which directly impact dopaminergic neurons, or indirectly affect the neurons by acting on other non-neuronal cells. Understanding the biomolecules mediating the cell-to-cell interaction will be helpful for better treatment of PD.

DJ-1, a 189 amino acid protein, has been linked to an autosomal recessive form of PD, despite being first identified as an oncoprotein [3]. Till date, the cells and biomolecules involved in the pathogenesis of DJ-1 deficient-associated PD remain unclear. In addition, DJ-1 knockout (KO) mice display only a mild PD phenotype and have no considerable loss of dopaminergic neurons [4]. Observation from the KO mice suggests that additional genetic or environmental factors are required for the development of PD phenotype following the loss-of-function mutations in DJ-1. Indeed, increased dopaminergic neuronal loss has been demonstrated in 1-methyl-4-phenyl-1,2,3,6-tetrahydropyridine (MPTP)-treated DJ-1 KO mice [5], suggesting that neurotoxins are adjunct factors for the development of DJ-1 phenotypes. Similarly, neuronal cells with DJ-1 mutation have been shown to be more sensitive to oxidative stress [6, 7], indicating that oxidative stress is another factor for the expression of DJ-1 phenotypes.

In addition to neurotoxicity, DJ-1 deficiency also affects neuroinflammation, since knockdown or KO of DJ-1 can sensitize glia cells to various inflammatory stimuli to display pro-inflammatory phenotypes. For example, microglia cells and astrocytes with DJ-1 deficiency are sensitized to proinflammatory stimulation of dopamine and lipopolysaccharide (LPS), respectively [8, 9]. In addition, DJ-1 deficiency potentiates interferon (IFN)-γ-induced proinflammatory responses in cultured microglia cells and astrocytes [10]. Since neuroinflammation plays critical roles in the pathogenesis of PD [11], it can be predicted that DJ-1 deficiency-induced proinflammatory responses might lead to neuronal death. However, repeated intraperitoneal injection of low-dose LPS into DJ-1 KO mice does not increase the vulnerability of dopaminergic neurons [12]. Since the blood-brain barrier (BBB) may prevent the intraperitoneally injected LPS from reaching the glia cells in the substantia nigra [13], the relationship between DJ-1 deficiency, glial activation, and neuronal death remains unclear.

Microglia are the main inflammatory cells in the brain. Although activated microglia act as the first line of defense to destroy infectious organisms, they also release a variety of cytotoxic substances to directly damage neurons and cause neuronal death. The microglia-derived neurotoxic substances include proinflammatory factors, such as tumor necrotic factor (TNF)-α, interleukin (IL)-1β, IL-6, IL-2, IFN-γ, as well as reactive oxygen and nitrogen species [14–17]. Microglial activation in the substantia nigra has been considered as a hallmark of PD, suggesting that neuroinflammation and its mediators contribute to the death of dopaminergic neurons and the progression of PD [11, 18]. However, the profile of proinflammatory factors, especially the microglia-secreted cytokines, in the substantia nigra in DJ-1 KO mice is not clear.

To clarify the mechanism related to DJ-1 deficiency in PD, we examined the change in the levels of cytokines and chemokines in the substantia nigra of DJ-1 KO mice. We demonstrated that DJ-1 deficiency caused an inflammatory sensitization of microglia to LPS administration, which may explain the role of DJ-1 in the etiology of PD.

## Materials and Methods

### Local injection of LPS into the substantia nigra of wild-type and DJ-1 knockout mice

Male DJ-1 KO mice were kindly provided by Dr. Tak W. Mak (Toronto, ON, Canada) [5]. Male wild-type (WT) C57BL/6 mice were used as controls and were obtained from the Animal Center of Medical College, National Taiwan University. The animals were housed in a temperature-controlled environment and allowed free access to food and water. The number of DJ-1 KO mice and WT mice in this study is 53 and 54, respectively. They were all 10–12 weeks-old and weighed 24–28 g. The animal experiments were performed in accordance with the National Institutes of Health (NIH) Guidelines on Laboratory Animal Welfare and approved by the Institutional Animal Care and Use Committee of the National Taiwan University.

The mice were anaesthetized with 200 mg/kg trichloroacetaldehyde and positioned on a stereotaxic apparatus (Narishige Scientific Instruments, Tokyo, Japan). One μL sterile LPS solution (1 μg/1 μL saline, Sigma, St. Louis, MO) [19] or sterile saline was locally injected into one side of the substantia nigra using a 2 μL Hamilton microsyringe (Stoelting, USA). The stereotaxic coordinates were 2.8 mm posterior to the bregma, 1.3 mm lateral to the midline, and 4.5 mm ventral to the surface of the dura mater [19]. The syringe needle was kept in place for 3 min and then pulled out. After anesthetic recovery, animals should be placed into a new clean cage and we took their body temperature and monitored basic biologic functions of intake, elimination and abnormal behavior of postoperative pain (attention to surgical site, decreased activity and abnormal posture). The recovery period in our studies may last from minutes to hours. In addition, we used antibiotics at surgical area to prevent contamination and reduce the risk of postoperative infection. Five days after the injection, the mice were sacrificed by carbon dioxide and the brains were harvested for immunohistochemical detection of proteins in the substantia nigra.

### Immunohistochemistry and double immunofluorescence staining

Mice were deeply anesthetized and transcardially perfused with 4% paraformaldehyde in saline; the brains were taken out and sequentially soaked in 4% paraformaldehyde and 30% sucrose solution. The brains were then cut into 30 μm coronal frozen sections. Before immunostaining, the tissue sections were pretreated with 3% hydrogen peroxide to inhibit the endogenous peroxidase activity, and incubated with 5% bovine serum albumin (BSA) and 0.1% Triton X-100 in phosphate-buffered saline (PBS) for 1 h for non-specific blocking and cell permeabilization, respectively.

For immunohistochemistry, the tissue sections were incubated with primary antibodies overnight at 4°C, and then incubated with the appropriate secondary antibodies for 1 h at room temperature. The primary antibodies used were rabbit anti-tyrosine hydroxylase (TH) (1:2500, Calbiochem Inc., San. Diego, CA, USA), mouse anti-Cd11b (1:500, AbD Serotec, Oxford, UK), rat anti-IFN-γ (1:500, abcam, Cambridge, UK), and goat anti-I-TAC (1:200, Santa Cruz, CA, USA) antibodies, while the secondary antibodies used were biotinylated secondary antibodies (1:500, Vector Laboratories, Burlingame, CA, USA). Finally, the tissue sections were treated with the avidin-biotin-peroxidase complex (ABC kit; Vector Laboratories. Burlingame, CA, USA) for 30 min, and the antibody-bound proteins were visualized by incubating with 0.01% hydrogen peroxide and 0.05% 3,3'-diaminobenzidine (DAB; Sigma, St. Louis, MO). The TH-positive or CD11b-positive cells were automatically identified using a public software ImageJ (National Institutes of Health; http://rsbweb.nih.gov/ij/), and their summated number were estimated by cumulatively counting the TH-positive or CD11b-positive cells every sixth section from the rostral end to the caudal end of the substantia nigra [20–22].

Double immunofluorescence staining were performed to check the colocalization of the protein with the microglia marker Cd11b. The brain sections were stained with the primary mouse anti-Cd11b (1:500, AbD Serotec, Oxford, UK) antibody, together with either rat anti-IFN-γ (1:500, abcam, Cambridge, UK) or goat anti-I-TAC (1:200, Santa Cruz, CA, USA) antibodies. Next, the tissues were incubated with mixed secondary antibodies, comprising of Alexa Fluor 546-conjugated anti-mouse antibody (1:500, Invitrogen, Carlsbad, CA), together with Alexa Fluor 488-conjugated anti-rat or anti-goat antibody (1:500, Invitrogen, Carlsbad, CA, USA). Fluorescence images were captured using a confocal microscope (Leica TCS SP2, Heidelberg, Germany).

### High-performance liquid chromatography

To evaluate the levels of dopamine and dopamine metabolites in the striatum, fresh striatal tissues were subjected to high-performance liquid chromatography (HPLC) analysis. The striatum of mice was dissected from the cerebral hemisphere, weighed, and homogenized in 0.1 N perchloric acid by sonication. For removing tissue debris, the homogenates were centrifuged at 14,500 *g* for 30 min at 4°C, and the remaining supernatant was then directly injected into a HPLC machine (Waters, Milford, MA) coupled with an electrochemical detector. Peaks of dopamine, dihydrophenylacetic acid (DOPAC), or homovanillic acid (HVA) were identified in the chromatograms by using the information of elution time of monoamine standards (Sigma, St. Louis, MO). Levels of dopamine, DOPAC, and HVA were then estimated by calculating their peak areas in the chromatogram, and were calculated as % of mean value in saline-treated WT mice.

### Multiplex array analysis

Profiling of cytokines and chemokines was conducted using a multiplex cytokine array. Briefly, protein extracted from the substantia nigra of WT and DJ-1 KO mice was quantified by using a BCA protein assay kit (Pierce, Rockford, IL), followed by analysis with a mouse Cytokine Array Panel A kit (R&D Systems, Minneapolis, MN, USA) according to the manufacturer’s instruction. Proteins (200 μg / array) were loaded onto the surface of the arrays for binding the primary antibodies coated on the array surface, washed to remove the unbound proteins, and incubated with HRP-conjugated secondary antibodies to form sandwich complexes. After that, the array membranes were washed again and the arrays were developed using an enhanced chemiluminescence (ECL) substrate. The array images were then captured by a charge-coupled device (CCD) imaging and documentation system (UVP, Upland,. CA, USA). Levels of protein spots in both WT and KO groups were measured by using ImageQuant 5.0 software, and normalized against the corresponding protein spots in the WT group.

### Bioinformatics analysis

To reveal the hub protein in the cytokine network regulated by DJ-1 (PARK7), the bioinformatics tool STRING (Search Tool for the Retrieval of Interacting Genes/Proteins) was used [23]. The networks were generated by algorithmically assembling the differentially expressed inflammatory molecules on the arrays and their interacting proteins from the STRING database.

### Primary mixed glial cultures derived from WT and DJ-1 knockout mice

For checking the effect of DJ-1 deficiency on the response of glial cells without the interactive influence of nearby neurons, a primary mixed glial culture was prepared from WT and DJ-1 KO mice using a previously described protocol [24]. Briefly, the whole brains of 52 1-day-old mice (WT and DJ-1 KO) were dissected and dissociated. The cells were maintained in T75 flasks in a humidified incubator (5% CO_2_, 37°C), and in Dulbecco’s modified Eagle’s medium (DMEM) containing 10% heat-inactivated fetal bovine serum (FBS) (Biological Industries, Kibbutz Beit Haemek, Israel), 100 U/mL penicillin and 0.1 mg/mL streptomycin. Fourteen days later, the cells were trypsinized and changed to 6-well culture plates for further experiments. The cultures contained 70% astrocytes stained with GFAP antibody (Santa Cruz, CA, USA) and 30% microglia stained with CD11b antibody (Serotec, Oxford, UK).

The primary mixed glial cells were seeded onto 6-well plates with DMEM medium at a density of 3 × 10^5^ cells/well, and the seeded cells were then cultured in serum-free medium for 16 h. Serum-starved glia cells were treated with LPS at a concentration of 100 or 300 ng/mL for a fixed period of time [25, 26]. After 24 h (for mRNA) or 48 h (for protein) culture, the cells and media were separately collected; the cells were lysed and subjected to quantitative real-time RT-PCR for measuring mRNA expression of IFN-γ or I-TAC, while the culture supernatant was subjected to enzyme-linked immunosorbent assay (ELISA) to measure extracellular protein concentration of IFN-γ or I-TAC. Finally, the levels of IFN-γ and I-TAC were compared between LPS-treated glia cells derived from WT and DJ-1 KO mice [27].

### Stable transfection of BV-2 cells with DJ-1 shRNA

The murine BV-2 cell line is an alternative cell model reproducing the characteristics of primary microglia with high fidelity [28, 29] and was a gift from Dr. J.S. Hong (NIEHS, NIH, NC, USA). BV-2 cells were maintained in DMEM medium supplemented with 10% heat-inactivated FBS, 100 U/mL penicillin and 0.1 mg/mL streptomycin (Invitrogen, Carlsbad, CA). DJ-1 knockdown was achieved by RNA interference. Briefly, BV-2 cells were cultured in a standard 6-well plate and transfected with a plasmid expressing shRNA to target DJ-1 transcript (Target Sequence: ATCTGGGTGCACAGAATTTAT) (Academia Sinica, Taipei, Taiwan) or empty vectors (pLKO.1; abbreviated as PLKO) as a control by using Oligofectamine reagent (Invitrogen, Carlsbad, USA) dissolved in OPTI MEM medium. Six hours after transfection, culture medium was changed back to DMEM medium, and puromycin (1 ng/mL) was added into the cultured medium 48 h later to kill cells without chromosomal integration of the gene. A stably transfected pool was selected after cell culture in puromycin-containing medium for several weeks, and knockdown of DJ-1 were confirmed by Western blot analysis of DJ-1 protein.

To compare the LPS-induced microglia responses in the WT and DJ-1 knockdown conditions, we investigated the NF-κB activation and cytokine expression in BV-2 cells stably transfected with empty and DJ-1 shRNA vectors. Western blot analysis and luciferase reporter assay were performed for respectively analyzing NF-κB signaling and activity of NF-κB promoter, while quantitative real-time RT-PCR and ELISA were performed to respectively check the expression and secretion of cytokines, including IFN-γ and I-TAC. Both WT and gene knockdown cells were treated with LPS at a concentration of 300 ng/mL [26], and cells without treatment with LPS were used as a control. In order to investigate the role of NF-κB in LPS-induced microglia responses, BV-2 cells were pre-treated with two NF-κB inhibitors for 1 h prior to LPS treatment. The inhibitors used were pyrrolidine dithiocarbamate (PDTC), which blocks IκBα degradation, and JSH-23 (Sigma, St. Louis, MO), which blocks NF-κB nuclear translocation.

### Effect of transwell co-cultured microglia cells on neuronal cells

A transwell co-culture device (Millipore, MA, USA) was used to investigate the effect of DJ-1 knockdown microglia cells on neuronal cells. In this device, BV-2 microglia cells were seeded onto the upper transwell inserts, while SH-SY5Y cells, which belong to a dopaminergic neuroblastoma cell line, were seeded onto the lower plate wells. First, SH-SY5Y cells were seeded onto the lower plate wells at 1 × 10^5^ cells/well in growth medium supplemented with 10% FBS and consisting 1:1 mixture of Eagle’s minimum essential medium (MEM) and Ham’s F-12 medium. Twenty hours later, BV-2 cells were seeded onto the upper insert wells with 0.4 um pore size (MERCK Millipore, Billerica, MA) at a density of 1 × 10^5^ cells per well, and the insert wells were immersed into the medium of the plate wells to start a non-contact co-culture of upper-well BV-2 and lower-well SH-SY5Y cells. After 24 h of co-culture, the medium of the upper insert wells was replaced with serum-free media with or without 300 ng/mL LPS for another 18 h, while the medium in the lower plate wells was replaced with fresh serum-free culture media. For antibody neutralization experiments, an anti-IFN-γ or anti-I-TAC antibody was added to both upper and lower wells 1 h before the treatment with LPS (300 ng/mL, 18-h) in the upper wells. Finally, the viability of SH-SY5Y cells in the lower plate wells were assessed using 3-(4,5-dimethylthiazol-2-yl)-2,5-diphenyltetrazolium bromide (MTT) assay.

### Dual-luciferase reporter assay

Cultured BV-2 cells with or without DJ-1 knockdown were cotransfected with a NF-κB-driven firefly luciferase reporter plasmid (1 μg) and a plasmid (0.1 μg) constitutively expressing Renilla luciferase by using the Oligofectamine reagent (Invitrogen, Carlsbad, CA) dissolved in OPTI MEM medium. Six hours after co-transfection, the medium was changed to serum-free DMEM medium for another 18 h. After 24 h of co-transfection, the cells were treated with LPS (300 ng/mL) or vehicle for 4 h. The treated cells were then lysed by using the Passive Lysis Buffer (Promega, Madison, WI, USA), and equal amount of proteins (20–30 μg) of the lysate supernatant for each sample was sequentially mixed with two reagents for measuring the activities of firefly and Renilla luciferases. The luminescent signals was detected using a Dual-Luciferase Reporter Assay System (Promega, Madison, WI, USA), and the firefly luciferase activity value was normalized to that of Renilla luciferase activity, which is regarded as a control for transfection efficiency.

### Quantitative TagMan real-time RT-PCR assay

Total RNA was prepared from brain tissues or cultured cells using a guanidinium thiocyanate-phenol-chloroform extraction solution (MDBio Inc., Taipei, Taiwan). Messenger RNA was then converted to complementary DNA (cDNA) by using MMLV RTase (Promega Co., USA), and analyzed by quantitative TagMan real-time reverse transcription polymerase chain reaction (RT-PCR) by using TagMan assay kits (Applied Biosystems, Foster City, CA), each of which contains a pair of predesigned unlabeled PCR primers and one predesigned TaqMan probe. The following TaqMan gene expression kits were used: Mm01168134_m1 for mouse IFN-γ, Mm00444662_ml for mouse I-TAC, and Mm99999915_g1 for GAPDH as a housekeeping gene. Real-time RT-PCR was performed by using a StepOne real-time PCR system (ABI, USA). The expression levels of IFN-γ and I-TAC were normalized to GAPDH by using the 2^-ΔΔCT^ method and are presented as % control.

### Western blotting

Cells were lysed in RIPA buffer (150 mM NaCl, 50 mM Tris—HCl, 1 mM EGTA, 1% Nonidet P-40, 0.25% deoxycholate, 1 mM sodium fluoride, 50 mM sodium orthovanadate) supplemented with Halt protease inhibitor cocktail (Thermo, IL, USA). Protein concentration was determined by using the BCA protein assay kit (Pierce, Rockford, IL) with serially diluted BSA as standards. Proteins were separated by SDS-PAGE and transferred to PVDF membranes (Millipore, Billerica, MA). The blot membranes were then blocked with 5% skim milk in PBS buffer at room temperature for 1 h, followed by overnight incubation with primary antibodies at 4°C, and finally incubated for 1 h at room temperature with the appropriate HRP-conjugated secondary antibodies. The primary antibodies used were rabbit anti-IKKα/β, rabbit anti-IκB-α or mouse anti-p65 (1:1000; all from Santa Cruz, CA, USA), mouse anti-phospho-IkB-a (Ser32/36) or rabbit anti-phospho-IKKα/β (Ser176/180) (1:1000; both from Cell Signaling Technology, Danvers, MA, USA), rabbit anti-DJ-1 (1:3000; Enzo Life Sciences, UK), and mouse anti-β-actin (1:10,000; Millipore, MA, USA) antibodies. The secondary antibodies used were HRP-conjugated goat anti-rabbit or goat anti-mouse IgG antibodies (1:10,000; Genetex, CA, USA). The protein bands were visualized using an ECL detection kit (Thermo, IL, USA), and blot images were captured by a gel document system (UVP; Upland, CA, USA).

### Isolation of cytoplasmic and nuclear protein fractions

For preparation of the cytoplasmic and nuclear extracts, NE-PER Nuclear and Cytoplasmic Extraction Reagents (Thermo Scientific, Rockford, IL USA) were used. Briefly, BV-2 cells were cultured in 10-cm dishes, and treated with LPS. The cytoplasmic and nuclear fractions were separated by using the extraction kit according to manufacturer’s instructions. The protein fractions were then subjected to western blot analysis for determining NF-κB signaling.

### Enzyme-linked immunosorbent assay

For determining the IFN-γ and I-TAC concentrations in the conditioned media and animal tissues, ELISA assay was performed by using an ELISA kit (R&D Systems, Minneapolis, MN, USA) according to the manufacturer’s instructions.

### MTT assay

The cell viability was determined using MTT (Sigma, St. Louis, MO). In brief, the cells were incubated in the MTT solution (0.5 mg/mL) at 37°C for 30 min. After incubation, medium was removed from the plate wells, and dimethyl sulfoxide (DMSO) was added to the wells to completely dissolve the formazan crystals. Finally, the absorbance of the dissolved formazan was measured using a spectrophotometer set at 570 nm.

### Cell counting by the methylene blue assay

The methylene blue assay was performed as reported previously [30]. We conducted the experiment as follows: Conditional medium in the 24-well culture plate was removed and each well was rinsed using PBS. Methylene blue solution (200 μL; 98.15% HBSS + 1.25% glutaraldehyde + 0.6% methylene blue) was added to each well. After 37°C incubation for 60 min, the methylene blue solution was removed, and each well was rinsed with distilled water six times with gentle submergence. Subsequently, elution solution (400 μL; 50% ethanol + 49% PBS + 1% acetic acid) was added to each well. On a plate rotator at room temperature for 15 min, the elution solution was centrifuged at 12,000 g for 3 min. The solution (200 μL each well) was transferred to a 96-well plate and read by a microplate reader at 570 nm. A standard curve was established by a range of cell densities of the cells in the culture plate. Absorbance values were converted to cell numbers using an equation of the line of the standard curve.

### Midbrain neuron-glia mixed cultures

To evaluate the neurotoxic effects of LPS, IFN-γ, and I-TAC in the presence of glia cells, the midbrain neuron-glia mixed cultures from 48 E14 rats were prepared as reported previously with some modifications [31]. Briefly, the ventral midbrain tissues from SD rat embryos were dissected in Hank balanced salt solution (GIBCO, Grand Island, NY) and dissociated by passage through a Pasteur pipette. The cells were then seeded on poly-D-lysine-coated 24-well culture plates at 3.5 × 10^5^ /well in DMEM medium supplemented with 10% FBS, 100 U/mL penicillin, and 0.1 mg/mL streptomycin, and kept at 37°C in a humidified atmosphere of 5% CO_2_. Twenty hours later, the medium was changed to MEM (GIBCO, Grand Island, NY) supplemented with 2% FBS, 2% horse serum, 100 U/mL penicillin, and 0.1 mg/mL streptomycin. Seven days later, the cells were treated with 500 ng/mL LPS (Sigma, St. Louis, MO) [32, 33], recombinant IFN-γ or I-TAC (R&D Systems, Minneapolis, MN, USA) for 48 h. Immunocytochemistry was performed to check the number of TH-positive neurons in the mixed cultures, and the survival of neurons undergoing different treatments were presented as % total number in the control group.

### Midbrain neuron-enriched cultures

To evaluate the neurotoxic effects of IFN-γ and I-TAC in the absence of glia cells, midbrain neuron-enriched cultures of E14 rat were prepared as reported previously with some modifications (Mount et al., 2007). Briefly, the ventral mesencephalic tissues of the rat brain were removed and then dissociated using a Pasteur pipette. Cells at 3.5 × 10^5^ /well were seeded on pre-coated poly-D-lysine 24 wells and kept in a 37°C humidified atmosphere of 5% CO2 and the maintenance medium consisted of DMEM with 10% FBS, 100 U/mL penicillin, and 0.1 mg/mL streptomycin. Twenty hours later, the maintenance medium was changed to serum-free MEM supplemented with 2% B27 supplement (Gibco), 100 U/mL penicillin, and 0.1 mg/mL streptomycin. Serum free-medium and B27 supplement can suppress glial-cell proliferation and promote neuronal-cell growth, respectively, thus enriching the neuron population. Seven days later, cells were treated with recombinant IFN-γ or I-TAC for 48 h, and then subjected to immunocytochemistry for measuring the TH-positive cells as described above.

### Statistics

All data are presented as the mean ± S.E.M. Student’s t-test was used for the statistical analysis between two samples. One-way analysis of variance (ANOVA) followed with Bonferroni’s post hoc test was used for statistical comparisons of more than two groups. Differences were considered significant as P < 0.05.

## Results

### Comparison of cytokine/chemokine expression profiles in the substantia nigra between WT and DJ-1 KO mice

**Fig 1:**
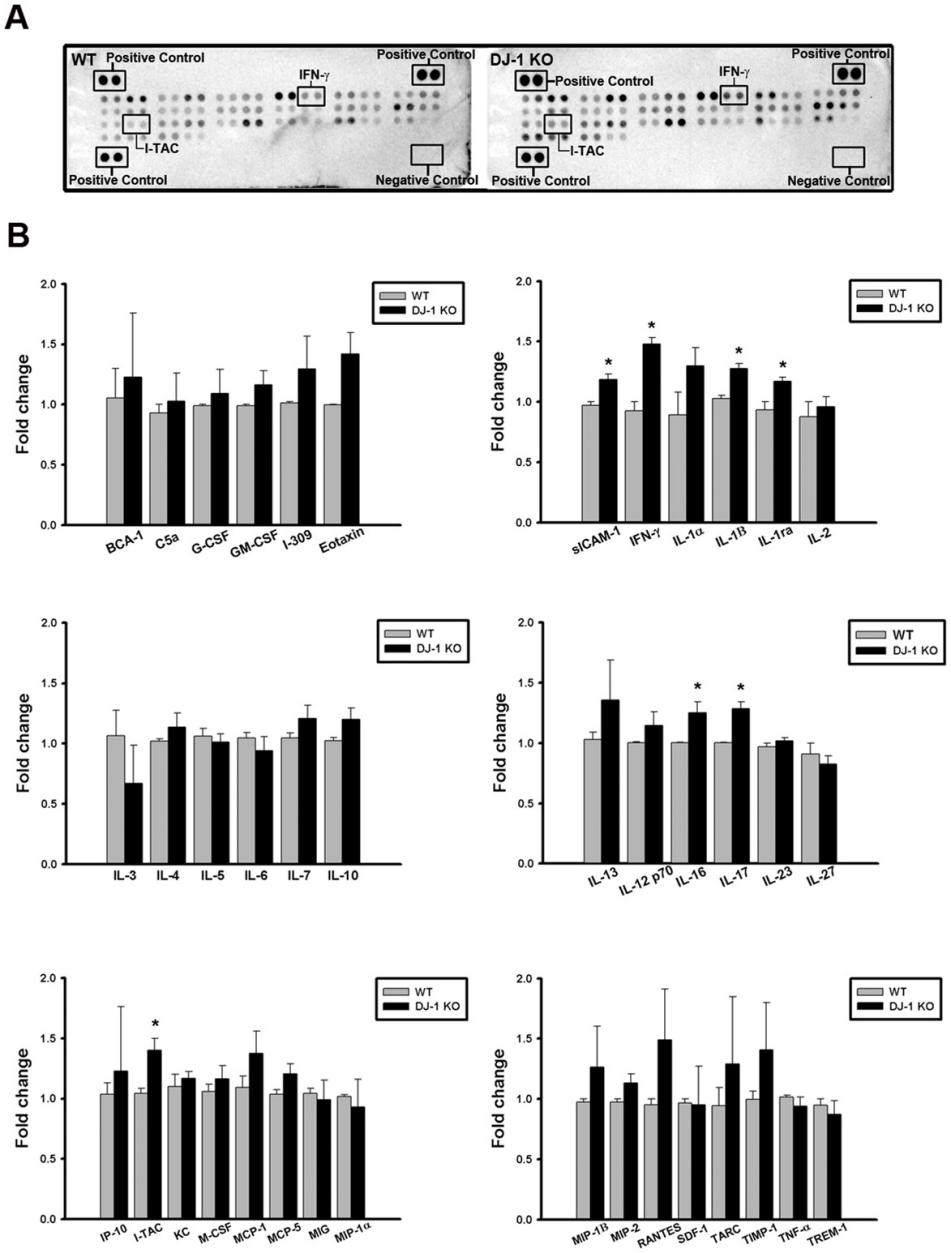
Increase of cytokines/chemokines expression in the substantia nigra of DJ-1 KO mice. (A) Cytokine arrays were used to evaluate the cytokine/chemokine contents. Representative array images showed the cytokine and chemokine profiles of substantia nigra protein extracts (200 μg) in WT (left) and DJ-1 KO (right) mice. (B) Bar charts showed the protein levels of WT and DJ-1 KO groups. Data were normalized as fold change of the respective protein spots in WT mice and presented as mean ± S.E.M. (n = 3 for each group) * p<0.05.

To determine the inflammatory proteins responsible for DJ-1 associated sensitization of the substantia nigra to LPS, we used protein arrays to examine the basal expression profiles of cytokines/chemokines in the substantia nigra of WT and DJ-1 KO mice. Our results showed that the cytokine-array profiles of the substantia nigra in WT (left of Fig 1A) and DJ-1 KO (right of Fig 1A) were different and showed a significant up-regulation of sICAM-1, IFN-γ, IL-1β, IL-1Ra, IL-16, IL-17, and I-TAC levels in DJ-1 KO mice as compared with those in WT mice (Fig 1B). These results indicate that the DJ-1 deficiency activates a mechanism leading to the up-regulation of particular inflammatory cytokines/chemokines in the substantia nigra.

### IFN-γ serves as a hub protein to regulate DJ-1 associated cytokine/chemokine network

**Fig 2:**
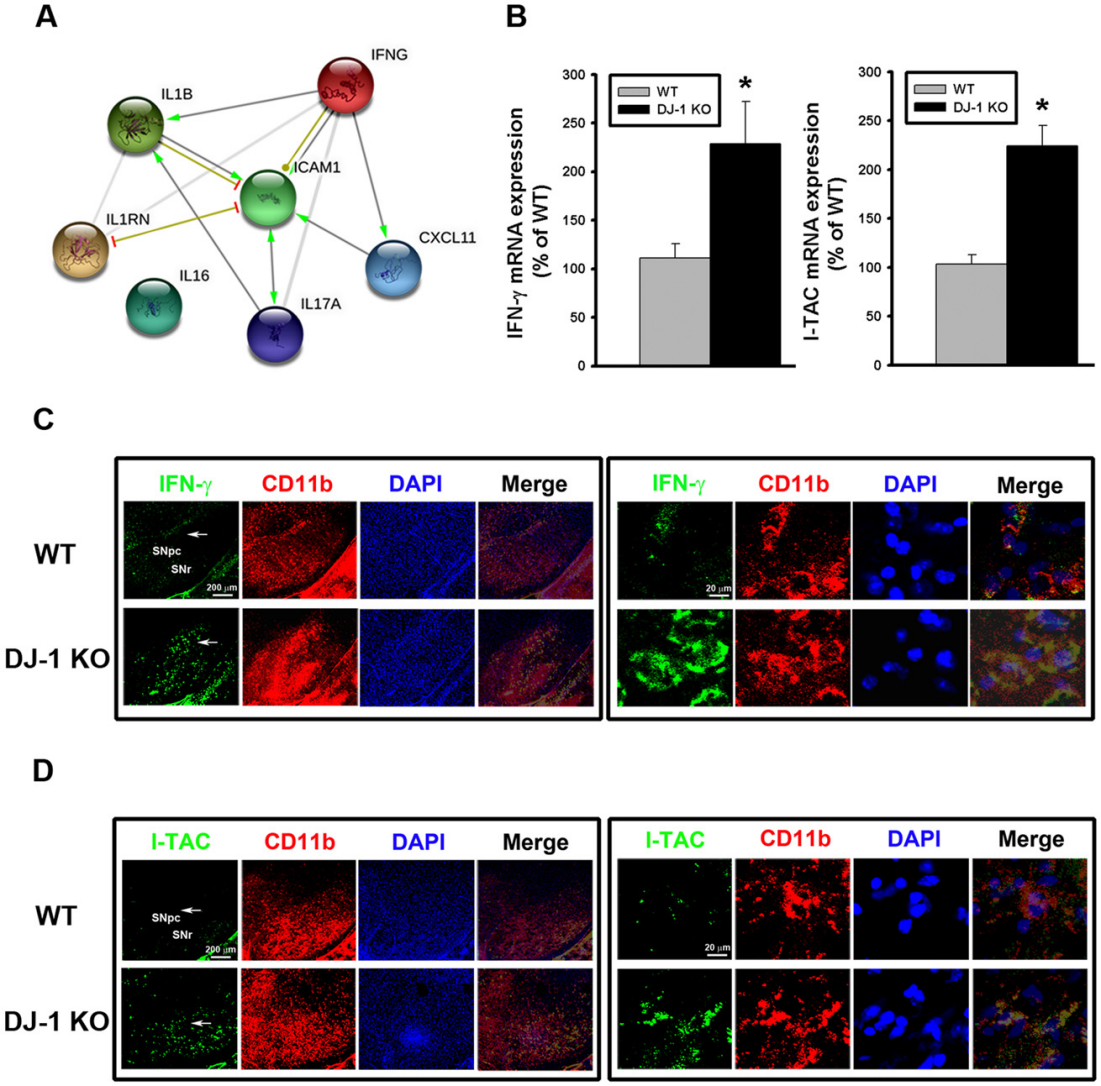
Up-regulation of IFN-γ and I-TAC in DJ-1 KO mice. (A) IFN-γ-regulated biological network predicted from the bioinformatics tool STRING. IFNG (i.e. IFN-γ) was predicted to serve as a hub protein to control CXCL11 (i.e. I-TAC), IL-17, IL-1β, IL1RN, and ICAM1. (B) The basal mRNA levels of IFN-γ (left panel) and I-TAC (right panel) in substantia nigra were up-regulated in DJ-1 KO mice as compared with those in WT mice. (C) Increase of basal IFN-γ expression in microglia in substantia nigra of DJ-1 KO mice. The immunofluorescent labeling images of IFN-γ (green), CD11b (red) and DAPI (blue) in substantia nigra of WT (upper images) and DJ-1 KO (lower images) mice were shown. (D) Increase of basal I-TAC expression in microglia in substantia nigra of DJ-1 KO mice. The immunofluorescent labeling images of I-TAC (green), CD11b (red) and DAPI (blue) in substantia nigra of WT (upper images) and DJ-1 KO (lower images) mice were shown. The arrow indicates the brain area that is magnified and shown in the right panel. The merged image shows the co-localization of two proteins in microglia. Data were normalized as percentage of mean mRNA level in WT mice and presented as mean ± S.E.M. (n = 5 for each group) * p<0.05. SNpc: substantia nigra pars compacta; SNr: substantia nigra pars reticulate.

For understanding the causal relationships among the cytokines and chemokines, we further performed bioinformatics analysis of the differentially expressed proteins by using STRING tool and database [23]. As shown in Fig 2A, the bioinformatics analysis revealed that IFN-γ is the key upstream protein (a hub protein), which can directly regulate downstream IL-1β, ICAM1, and I-TAC (i.e. CXCL11) and indirectly regulate other cytokines (Fig 2A). Among them, IL-1β and ICAM have been known to be regulated by DJ-1 [9, 34], while I-TAC is a novel target that has never been reported to be associated with DJ-1 deficiency. We further performed more in vivo and in vitro experiments to examine the role of IFN-γ (the hub cytokine) and I-TAC (the downstream mediator) in the development of DJ-1 associated phenotype, i.e. increased loss of dopaminergic neurons.

To further explore whether DJ-1 regulates the transcription of IFN-γ and I-TAC genes, TagMan real-time RT-PCR was performed to investigate their mRNA expressions in the substantia nigra. As shown in Fig 2B, mRNA levels of IFN-γ (left panel) and I-TAC (right panel) were significantly higher in DJ-1 KO mice as compared with WT mice, suggesting that the upregulation of IFN-γ and I-TAC expressions in DJ-1 KO condition is at the transcriptional level.

Since IFN-γ is a type II interferon known to be restrictedly expressed in immune cells, we performed a double immunofluorescence staining to check whether IFN-γ is located in microglia, which are resident immune-modulating cells in the brain. As shown in Fig 2C, fluorescence of IFN-γ (green) and CD11b (red) co-localized in the same cells, indicating that IFN-γ is expressed in microglia in the substantia nigra. Similarly, fluorescence of I-TAC (green) and CD11b (red) also co-localized in the same cells (Fig 2D), suggesting that I-TAC is also expressed in microglia in the substantia nigra.

### LPS-induced death of dopaminergic neurons is enhanced in DJ-1 KO mice

**Fig 3:**
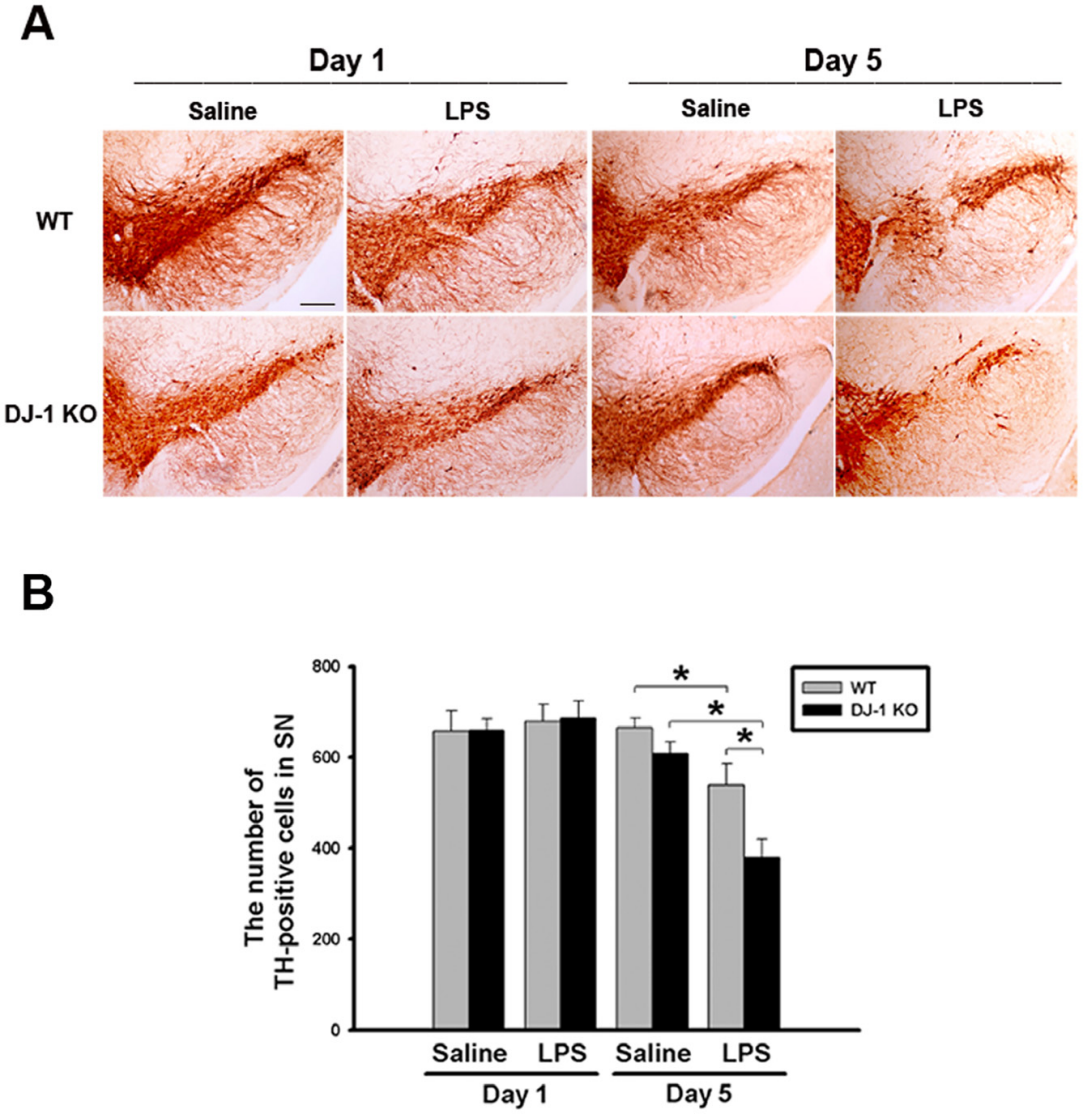
LPS-induced death of dopaminergic neurons is enhanced by local LPS administration in DJ-1 KO mice. LPS (1 μg /1 μl) or saline (1 μl) was locally injected into substantia nigra. One day and five days later, mice were sacrificed respectively. (A) Immunohistochemical images of substantia nigra showed the differential loss of TH-positive neurons in WT (upper panel) and DJ-1 KO (lower panel) mice Day 1 (left 4 images) or Day 5 (right 4 images) after local injection of LPS. Note that LPS-induced death of dopaminergic neurons was enhanced on Day 5 in DJ-1 KO mice. The statistical results were shown in (B). The mean of the summated neuron number was shown in each group and the data are presented as mean ± S.E.M. (n = 7 for each group) * p<0.05, Scale bar: 0.25 mm.

Since DJ-1 KO mice display only a mild PD phenotype and have no considerable loss of dopaminergic neurons [4], it is interesting to know whether the phenotypes of DJ-1 KO mice can be significantly changed by inflammatory stimuli, such as LPS [35]. We thus conducted the animal experiment at two time points (1 day or 5 days after LPS injection). Using immunohistochemistry (S1A Fig) and ImageJ software analysis (S1B Fig), it was found that microglial cells in the substantia nigra of WT and DJ-1 KO mice were activated 1 day after LPS injection compared with the individual control. The up-regulated level of CD11b, a β-integrin marker of microglia, represents microglial activation during neuroinflammation [36]. We also used Iba-1, another marker of activated microglia, to examine the microglial activation in WT and DJ-1 KO mice 1day after LPS treatment. The results (S1C and S1D Fig) showed that Iba-1 was also increased in the substantia nigra of WT and DJ-1 KO mice. However, there was no significant loss of dopaminergic neuronal cells (Fig 3, left 4 images) and change of TH level (S1C–S1E Fig) in WT and DJ-1 KO mice 1 day after LPS injection. In addition, there was a similar result in the striatum of WT and DJ-1 KO mice 1 day after LPS injection (S1F–S1H Fig). Notably, 5 days after the saline or LPS treatment, local injection of LPS into the substantia nigra caused a loss of TH-positive neurons by about 19% in WT mice and up to 43% in DJ-1 KO mice (Fig 3, right 4 images), as compared with saline injection. These results suggested that the inflammation-induced loss of dopaminergic neurons can be greatly augmented in the DJ-1 KO condition.

### LPS-induced reduction of dopaminergic fibers, dopamine content, and dopamine metabolites in the striatum of DJ-1 KO mice

**Fig 4:**
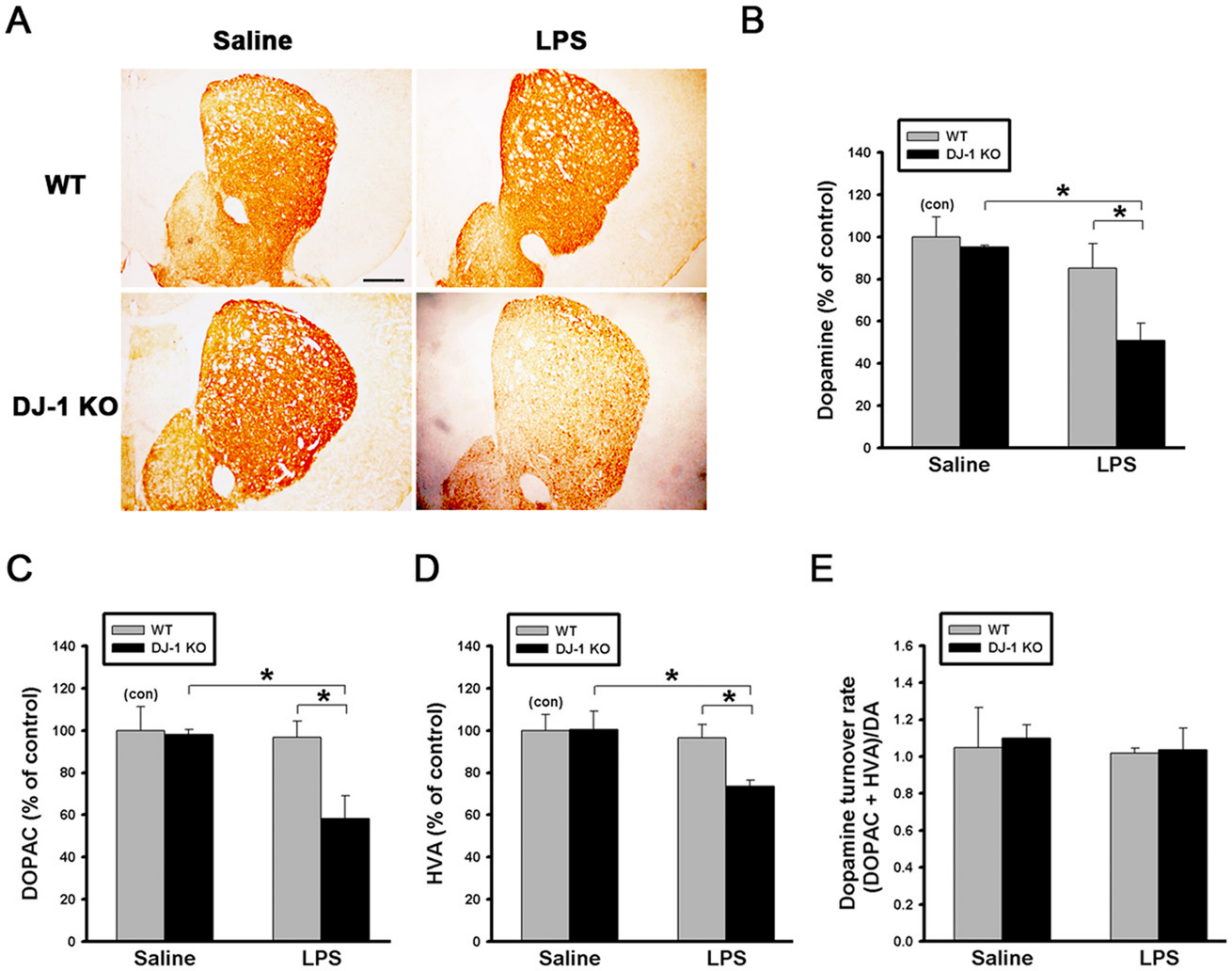
LPS-induced reduction of striatal dopamine content is enhanced by DJ-1 knockout. (A) LPS (1 μg /1 μl) or saline (1 μl) was locally injected into substantia nigra. Five days later, the mice were sacrificed. TH-positive nerve terminals in striatum were stained using immunohistochemistry, and (B) dopamine, (C) DOPAC and (D) HVA in striatum were measured by HPLC in WT and DJ-1 KO mice. (E) Turnover rates of dopamine in the striatum of WT and DJ-1 KO mice. The turnover rates were calculated by dividing summated levels of dopamine metabolites by corresponding levels of dopamine, and were used to evaluate the states of monoamine metabolism in dopaminergic neurons. Data were normalized as percentage of mean HPLC value in saline-treated WT mice (con) and presented as mean ± S.E.M. (n = 7 for each group) * p<0.05, Scale bar = 0.5 mm.

To examine whether the differential loss of TH-positive neurons (Fig 3) in the substantia nigra can lead to different dopamine deficiency in the striatum, we performed HPLC analysis to compare the levels of striatal dopamine and it metabolites in WT and DJ-1 KO mice 5 days after the saline or LPS treatment. Although the levels of TH-positive nerve terminals, dopamine, DOPAC, or HVA were similar (p > 0.05) between WT mice treated with saline and LPS (IHC staining of WT mice in Fig 4A, gray bars in Fig 4B–4D), our results showed that LPS can lead to a noticeable reduction of DJ-1 KO striatal dopaminergic fibers (IHC staining of DJ-1 KO mice in Fig 4A), and a remarkable reduction of striatal dopamine (45%, Fig 4B), DOPAC (32%, Fig 4C), and HVA (18%, Fig 4D) in DJ-1 KO mice (black bars) treated with LPS as compared with the saline control. The reduction of dopamine amount in the striatum is not due to an altered metabolic rate, since the dopamine turnover rate, which is calculated using the formula (DOPAC + HVA) / DA was not altered when the dopamine levels were reduced (Fig 4E). These results suggested that the LPS-induced loss of neurons was enhanced in the DJ-1 KO condition.

### LPS-induced expression / release of IFN-γ and I-TAC is enhanced by knockout of DJ-1

**Fig 5:**
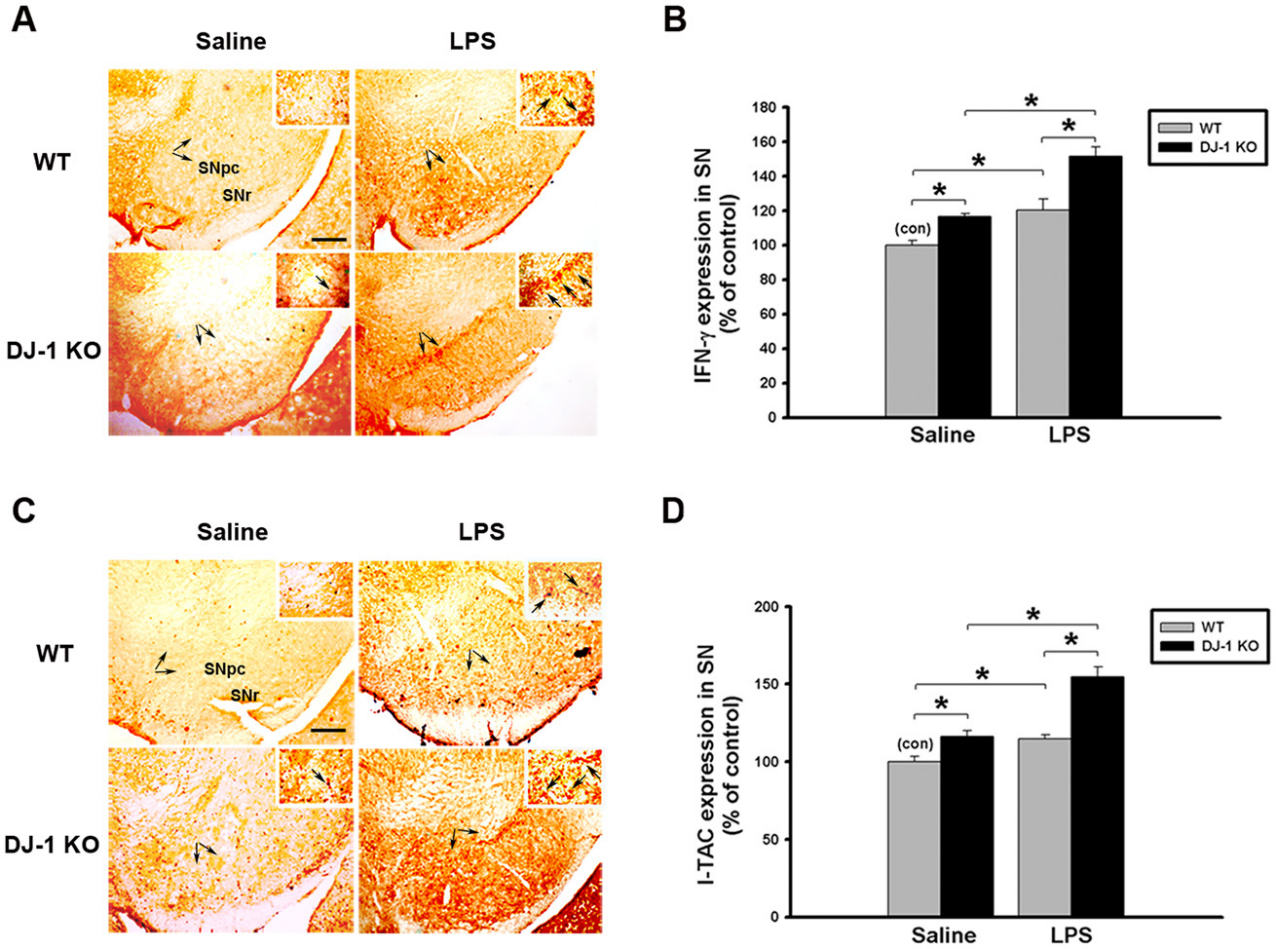
LPS-induced up-regulation of IFN-γ and I-TAC is enhanced in DJ-1 knockout mice. Levels of IFN-γ and I-TAC in substantia nigra (SN) were up-regulated by local injection with LPS (1 μg /1 μl) in DJ-1 KO mice. (A) Immunohistochemical staining of IFN-γ in substantia nigra of WT and DJ-1 KO mice Day-5 after injection of LPS. (B) Quantitative results (ELISA) show the increase of IFN-γ expression in substantia nigra of DJ-1 KO mice. (C) Immunohistochemical staining of I-TAC in substantia nigra of WT and DJ-1 KO mice Day-5 after injection of LPS. (D) Quantitative results (ELISA) show the up-regulation of I-TAC expression in substantia nigra of DJ-1 KO mice. The typical stained-cells were indicated by arrows. Data were normalized as percentage of the mean of basal expressional levels in WT mice (con) and presented as mean ± S.E.M. (n = 4–5 for each group) * p<0.05. SNpc: substantia nigra pars compacta; SNr: substantia nigra pars reticulate. Scale bar: 0.2 mm.

Since the LPS-induced loss of dopaminergic neurons was enhanced in DJ-1 KO mice, we investigated whether the LPS-induced expression of IFN-γ and I-TAC in the substantia nigra and cultured glia cells can be affected by knockout of DJ-1. First, we examined the effect of DJ-1 KO on IFN-γ and I-TAC expressions in the substantia nigra in vivo. In consistence with our previous results in KO mice shown in Figs 1 and 2, levels of both IFN-γ (Fig 5A, IHC staining; 5B, protein levels) and I-TAC (Fig 5C, IHC staining; 5D, protein levels) in the substantia nigra in Day-5 saline-treated group were higher in DJ-1 KO mice than those in WT mice and there was a slight increase of IFN-gamma and I-TAC in DJ-1 KO mice 1 day after LPS injection (S2 Fig) as compared with those on Day-5 after LPS injection (Fig 5). Therefore, we found that local application of LPS to the substantia nigra up-regulated both genes, which was further enhanced in DJ-1 KO mice as compared with WT mice.

**Fig 6:**
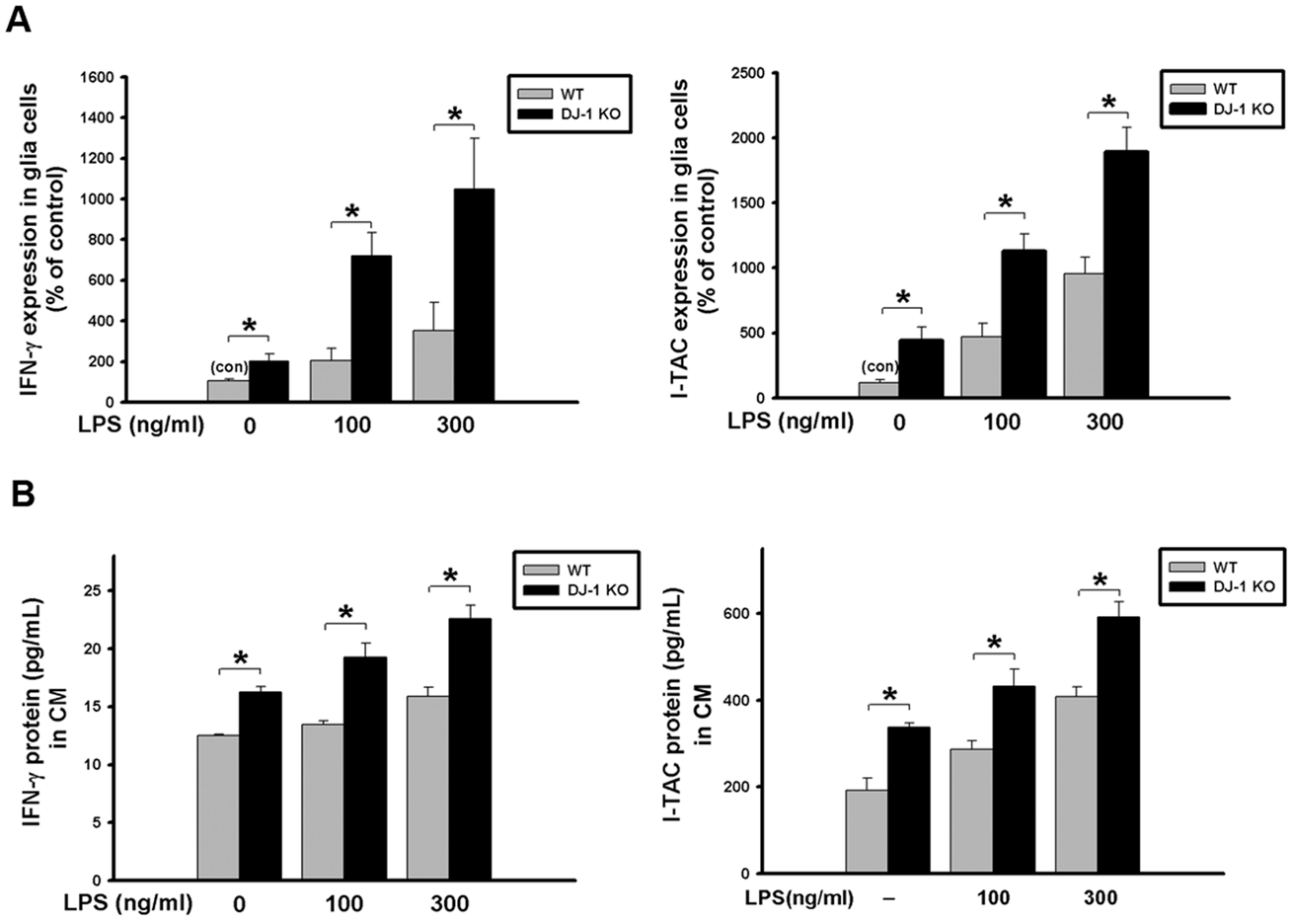
LPS-induced increase of IFN-γ and I-TAC is up-regulated in DJ-1 knockout mixed-glia cultures. Primary mixed glia cultures were derived from the brain of 1-day-old WT and DJ-1 KO mice. (A) The mRNA levels of IFN-γ and I-TAC measured by quantitative real-time RT-PCR were higher in DJ-1 KO cells than WT cells following LPS administration (100 and 300 ng/ml) for 24 hours. (B) The protein levels of IFN-γ and I-TAC measured by ELISA were increased in conditioned medium (CM) of DJ-1 KO cells as compared with WT cells following application of LPS (100 and 300 ng/ml) for 48 hours. Data were normalized as percentage of the mean of basal expressional levels in WT mice (con) and presented as mean ± S.E.M. (n = 4–5 for each group) * p<0.05.

Next, we isolated the neonatal brain from WT and DJ-1 KO mice and prepared mixed glia cultures. Similarly, the basal expression of IFN-γ and I-TAC in cultured glia cells from DJ-1 KO mice was significantly higher than that from WT mice (Fig 6A). In addition, protein concentrations of IFN-γ or I-TAC in the conditioned medium (CM) from DJ-1 KO glia cells were also higher than those from WT cells (Fig 6B), indicating that DJ-1 KO glia cells released more IFN-γ and I-TAC than WT cells in the basal condition. After the LPS challenge, the expression and release of either IFN-γ or I-TAC were further enhanced in the DJ-1 KO condition.

### NF-κB pathway is involved in the DJ-1 deficiency-induced up-regulation of IFN-γ and I-TAC

**Fig 7:**
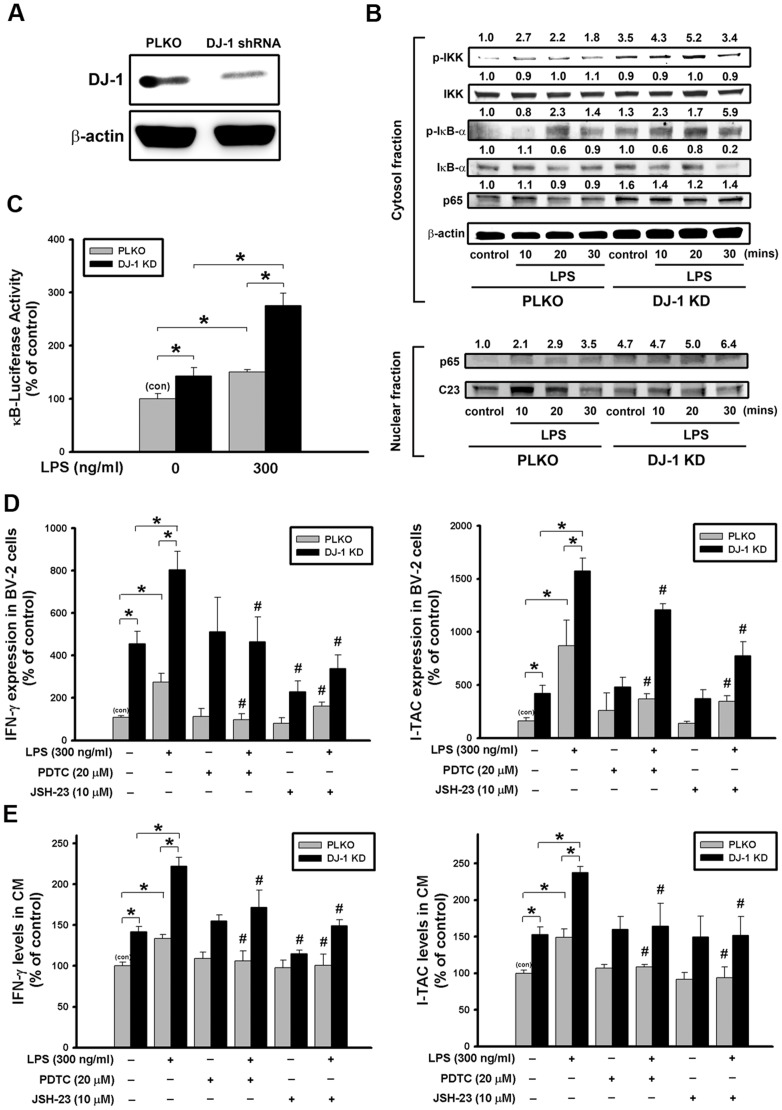
DJ-1 knockdown increases LPS-induced cytokine expression in BV-2 microglia cells via NF-κB pathways. (A) Western blots showed the knockdown of DJ-1 in BV-2 cells by stably transfecting cells with plasmids encoding DJ-1 shRNA as compared with control PLKO vector. (B) Immunoblotting showed that treatment of LPS (300 ng/ml) increased the phosphorylation of IκB-α, degradation of IκB-α and p65 nuclear translocation, which was enhanced in DJ-1 knockdown BV-2 cells. (C) Luciferase reporter assay showed that LPS stimulation for 4 hours enhanced the NF-κB promoter activity in DJ-1 knockdown cells. (D) mRNA expression (6 hours after LPS) and (E) protein secretion levels (24 hours after LPS) of IFN-γ and I-TAC were increased by LPS in BV-2 cells and conditioned medium (CM) respectively, and were further enhanced by DJ-1 knockdown. The elevated expression and secretion of cytokine can be antagonized by treating cells with NF-κB inhibitors PDTC and JSH-23. Data were normalized as percentage of mean basal level in group of control vector (con) and presented as mean ± S.E.M. (n = 4–5 for each group) * p<0.05, # p<0.05 as compared between conditions with and without inhibitor treatment, which were either with or without LPS stimulation.

Since IFN-γ and I-TAC were expressed in microglia in the substantia nigra as shown in Fig 2, we chose BV-2 as a microglia cell model to explore the mechanism underlying the DJ-1 knockdown-induced up-regulation of IFN-γ and I-TAC expressions. As shown in Fig 7A, DJ-1 protein expression was greatly suppressed in DJ-1 knockdown cells, indicating that a stable knockdown of DJ-1 has been successfully achieved in BV-2 cells.

Treatment of LPS increased the phosphorylation of IKK and IκBα, and causeed the ubiquitination and degradation of IκBα by proteasome [37]. We then examined whether the NF-κB pathway is involved in the LPS-induced up-expression of IFN-γ and I-TAC. As shown in Fig 7B and S3 Fig, DJ-1 knockdown cells showed earlier phosphorylation of IKK and IκBα and IκBα degradation than control cells after LPS treatment. Moreover, DJ-1 knockdown increased the p65 nuclear translocation under basal condition or after LPS treatment. We further examined the effect of DJ-1 knockdown on the NF-κB promoter activity 4 hours after LPS treatment. As shown in Fig 7C, the basal activity of the NF-κB promoter was higher in DJ-1 knockdown cells. LPS treatment further increased the promoter activity, which was greater in DJ-1 knockdown cells than in control cells (Fig 7C).

### DJ-1 Knockdown increases the LPS-induced expression / release of IFN-γ and I-TAC in BV-2 microglia

Since DJ-1 KO increased the expression/release of IFN-γ and I-TAC in primary glia cultures, we further examined whether DJ-1 knockdown affects IFN-γ and I-TAC expression/release from BV-2 cells. Our results showed that LPS-stimulated expression (Fig 7D) and secretion (Fig 7E) of either IFN-γ or I-TAC were higher in DJ-1 knockdown BV-2 cells than in control cells and could also be antagonized by NF-κB inhibitors PDTC and JSH-23. These results further demonstrated that DJ-1 knockdown enhanced the stimulatory effect of LPS in glial cells.

### IFN-γ and I-TAC increase the death of dopaminergic neurons in C57BL/6 mice

**Fig 8:**
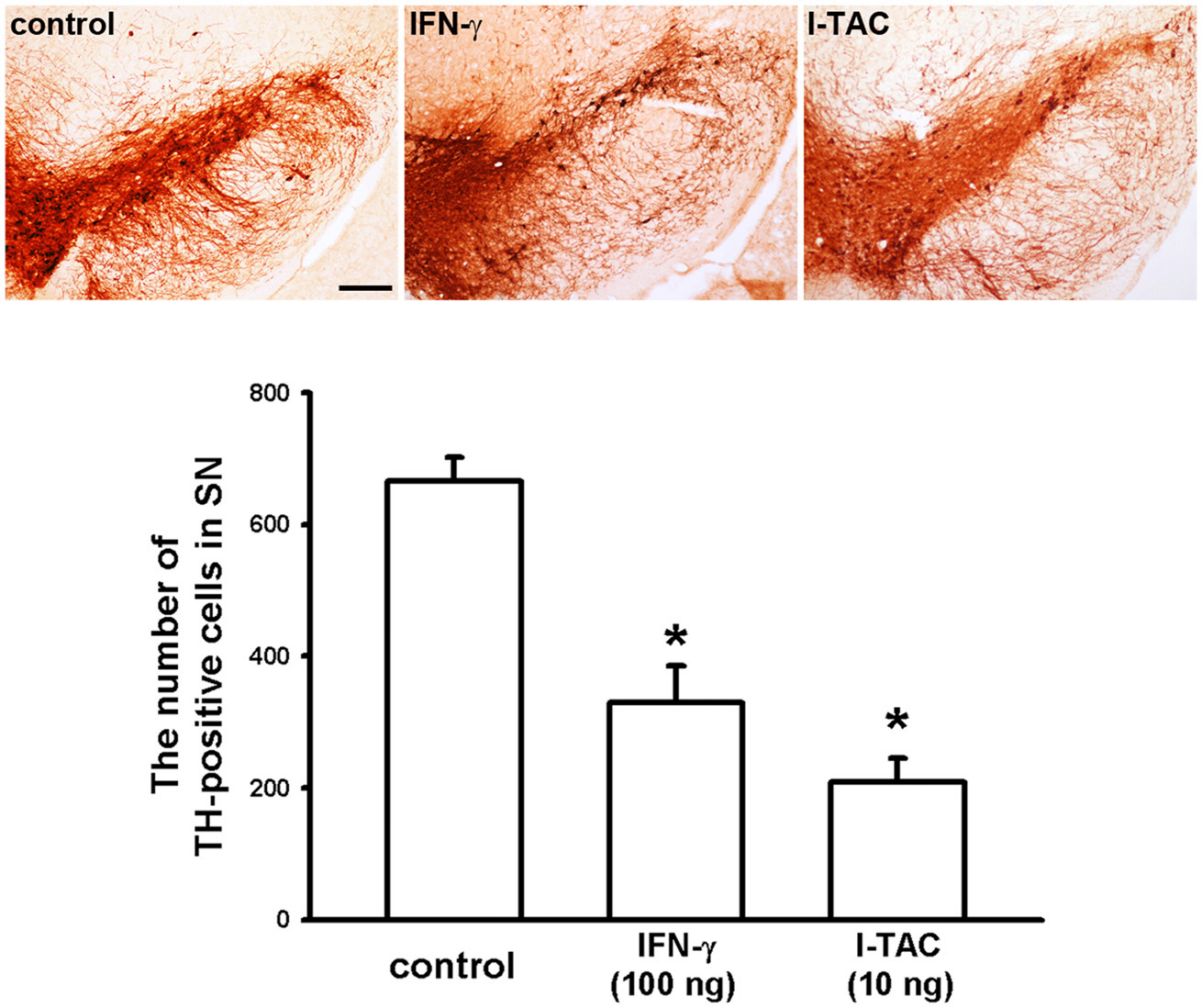
IFN-γ and I-TAC increase the death of dopaminergic neurons in substantia nigra of C57BL/6 mice. IFN-γ (100 ng /1 μl), I-TAC (10 ng /1 μl) or saline (control) was locally injected into substantia nigra. Five days later, C57BL/6 mice were sacrificed. Immunohistochemical images of substantia nigra showed the loss of TH-positive neurons in C57BL/6 mice following local injection of IFN-γ, I-TAC or saline. Note that IFN-γ- or I-TAC-induced death of dopaminergic neurons was increased in C57BL/6 mice as compared with control. The statistical results were shown below. Neuron number was mean number of TH-positive neurons in substantia nigra of each group and presented as mean ± S.E.M. (n = 5 for each group) * p<0.05, Scale bar: 0.2 mm.

To further examine whether IFN-γ or I-TAC could cause the loss of dopaminergic neuronal cells in vivo, we locally injected IFN-γ (100 ng/mL) or I-TAC (10 ng/mL) into the substantia nigra of C57BL/6 mice. We found that the number of dopaminergic neuronal cells was significantly reduced by intranigral administration of recombinant IFN-γ or I-TAC protein compared with control (Fig 8). Therefore, these experiments demonstrated the neurotoxic effect of IFN-γ or I-TAC on dopaminergic neurons in the substantia nigra.

### DJ-1 knockdown in BV-2 cells enhances the LPS-induced neuronal death in co-cultured SH-SY5Y-BV-2 cells

**Fig 9:**
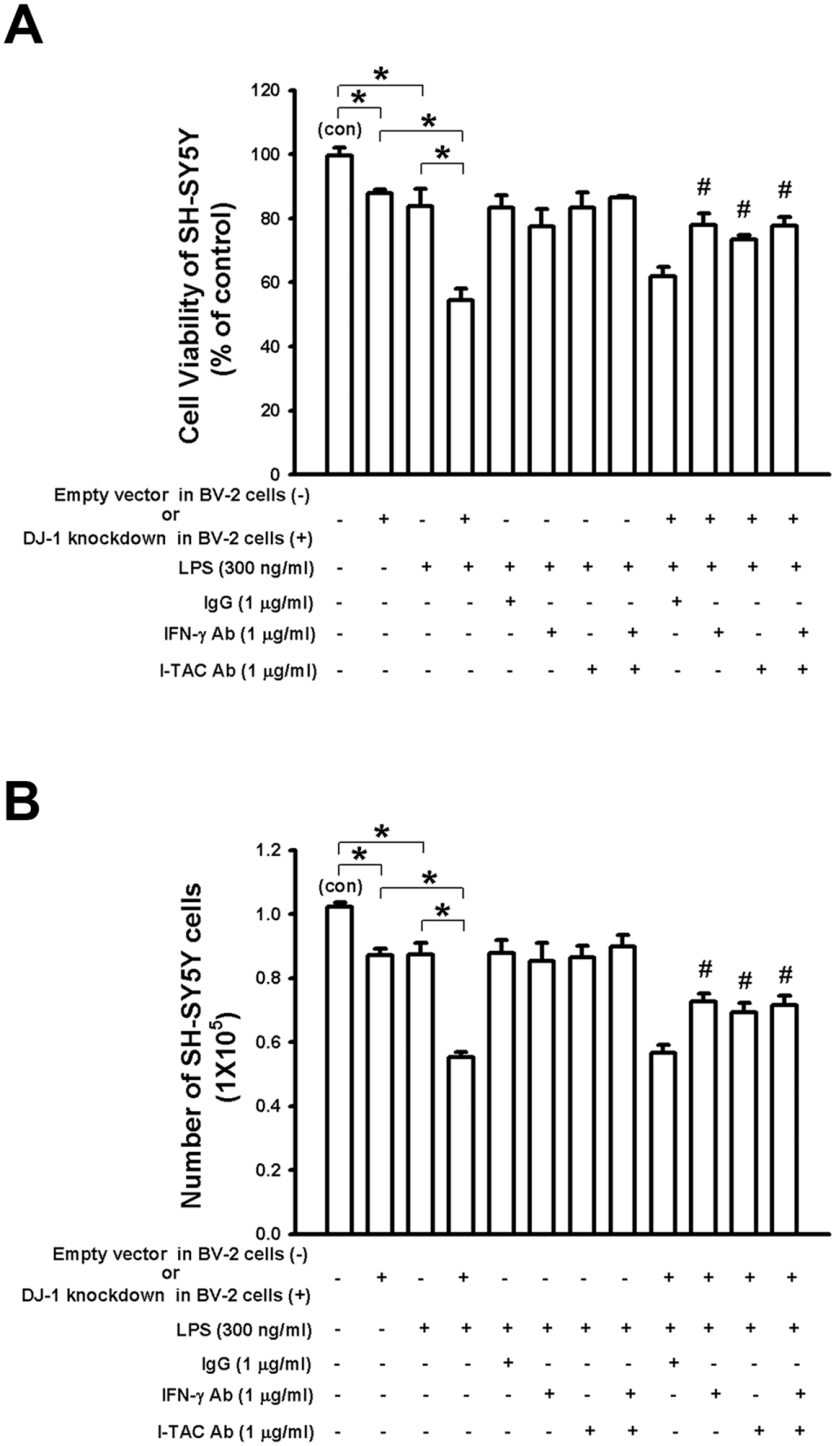
DJ-1 knockdown in BV-2 microglia cells enhances LPS-induced death of co-cultured SH-SY5Y neuronal cells. In the co-culture device, control vector- or DJ-1 shRNA-transfected BV-2 cells were cultured in the upper inserts, while SH-SY5Y cells were cultured in the lower plate wells. Cell viability of SH-SY5Y was evaluated by using MTT assay (A) and methylene blue assay (B). Note that LPS (300 ng/ml, for 18 hours) reduced viability of neuronal cells, which was further decreased by co-culture with DJ-1-knockdown BV-2 cells. The viability was markedly decreased in co-culture with DJ-1-knockdown BV-2 cells and LPS treatment, which can be antagonized by IFN-γ and/or I-TAC neutralizing antibody. Data were normalized as percentage of mean neuron number in the co-culture without any treatment (con) and presented as mean ± S.E.M. (n = 4–5 for each group) * p<0.05, # p<0.05 as compared with DJ-1-knockdown BV-2 cells with LPS treatment.

To further evaluate the effect of DJ-1 knockdown microglia on the survival of nearby neuronal cells, we used a transwell co-culture of BV-2 and SH-SY5Y cells, which is a well-established model for examining the cross-talk between different cells [38]. BV-2 cells were seeded in the upper chamber and SH-SY5Y cells were cultured in the lower chamber. The combination of DJ-1 knockdown and LPS treatment in BV-2 cells caused the greatest reduction in the viability of co-cultured SH-SY5Y neuronal cells, which was antagonized by IFN-γ and/or I-TAC antibody treatment (Fig 9A (MTT assay), 9B (Methylene blue assay).

### IFN-γ and I-TAC increase death of dopaminergic neurons in primary neuron-glia co-cultures

**Fig 10:**
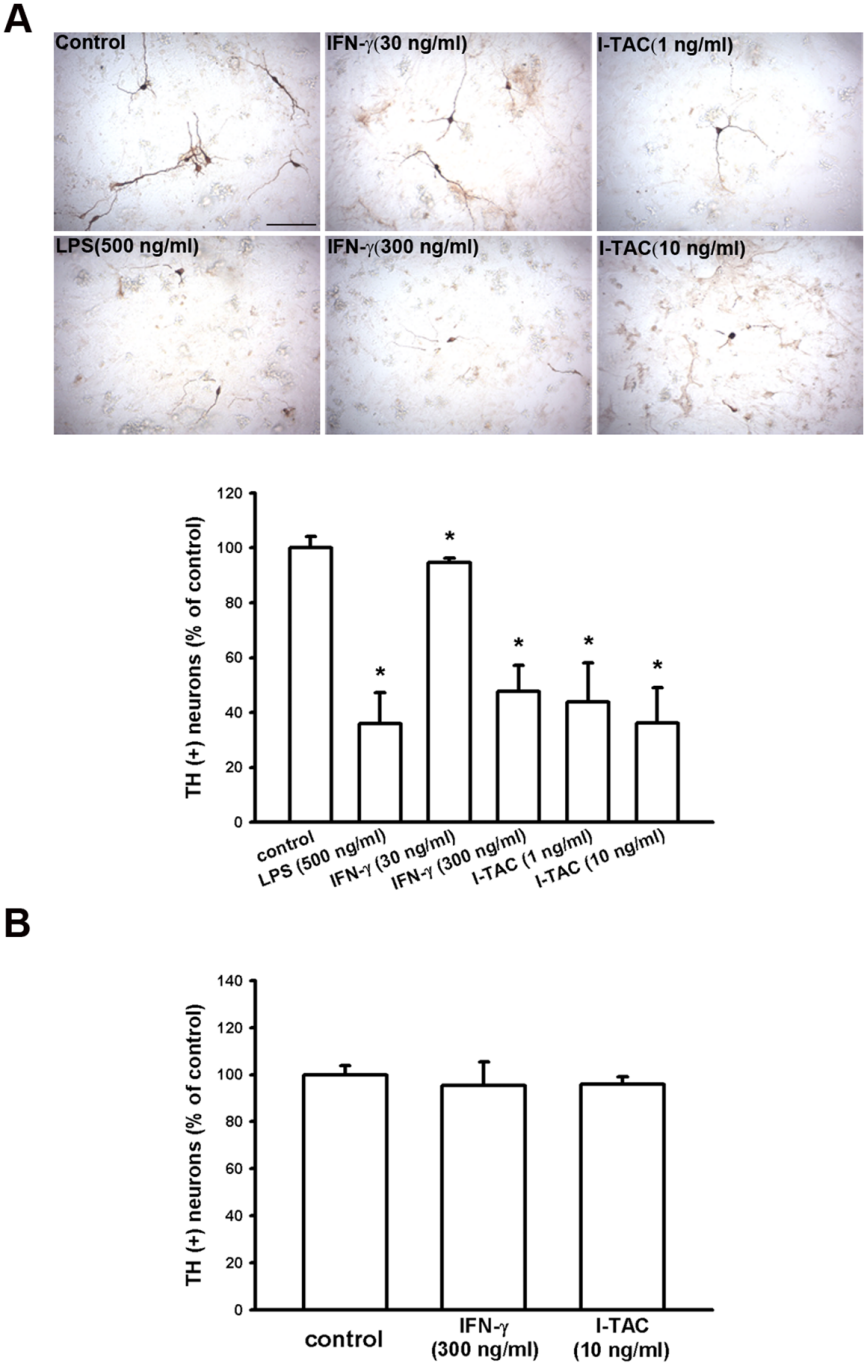
IFN-γ and I-TAC reduce the survival of dopaminergic neurons in primary midbrain neuron-glia mixed cultures but not in neuron-enriched cultures. (A) Midbrain neuron-glia mixed cultures were derived from E14 rat embryos and treated with recombinant IFN-γ protein (30 and 300 ng/ml) or I-TAC protein (1 and 10 ng/ml) on Day-7 cultures for 48 hours. LPS (500 ng/ml) was used as positive control. Note that IFN-γ and I-TAC reduced the survival of TH-positive neurons in neuron-glia mixed cultures (B) The neuron-enriched cultures were obtained from E14 rat embryos and treated with IFN-γ and I-TAC on Day-7 cultures for 48 hours. Note that IFN-γ and I-TAC did not affect survival of TH-positive neurons in neuron-enriched cultures. The dopaminergic neurons (TH-positive) were counted and normalized as percentage of TH-positive neurons in control cells. Data were presented as mean ± S.E.M. (n = 4–5 for each group) * p<0.05 as compared with control.

Since IFN-γ and I-TAC were involved in the neurotoxic activity in the substantia nigra of mice (Fig 8) and microglia (Fig 9), we then examined whether IFN-γ or I-TAC can affect the survival of dopaminergic neurons in either a primary neuron-glia co-cultures or neuron-enriched cultures. Our results showed that treatment of recombinant IFN-γ or I-TAC significantly reduced the number of TH-positive cells in neuron-glia co-cultures (Fig 10A), but not in neuron-enriched cultures (Fig 10B). Furthermore, we also found that there was no synergistic effect of IFN-γ and I-TAC in midbrain neuron-glia mixed cultures (S4 Fig). These differential effects suggest that IFN-γ and I-TAC indirectly caused the dopaminergic neuronal death through activation of glia cells.

## Discussion

In the present study, we demonstrated that the levels of IFN-γ and I-TAC in the substantia nigra were quite different between DJ-1 KO and WT mice (Fig 1). There was a differential loss of dopaminergic neurons in the Day-5 LPS-injected substantia nigra (Fig 3) and depletion of striatal dopamine (Fig 4) between WT and DJ-1 KO mice. Bioinformatics analysis further indicated that IFN-γ is a key hub protein to regulate other cytokines and chemokines, such as I-TAC, to mediate the DJ-1 deficiency-induced neuronal loss (Figs 2, 5 and 6). Moreover, in vitro experiments revealed that IFN-γ and I-TAC were up-regulated by DJ-1 deficiency through NF-κB signaling in LPS-treated microglia cells (Fig 7). IFN-γ and I-TAC released from microglia cells then led to the death of co-cultured neuronal cells, and intranigral injection of IFN-γ or I-TAC caused the loss of dopaminergic neurons (Figs 8, 9 and 10). These results may account for how a genetic factor (i.e. DJ-1) and an inflammatory agent (e.g. LPS) together contribute to the development of PD in a DJ-1 deficient condition.

Although DJ-1 is linked to early-onset PD, there is no obvious PD phenotype in DJ-1 KO mice [5]. Therefore, DJ-1 deficiency alone is not sufficient to cause PD. However, many in vitro studies have demonstrated that DJ-1 can lower the threshold for other factors to cause neuronal damage in a direct or indirect way. For example of a direct way, dominant negative mutation or KO of DJ-1 has been shown to sensitize neuronal cells to oxidative stress [6, 7] and to enhance neurotoxin-induced neuronal death [5]. For example of an indirect way, knockdown of DJ-1 has been revealed to potentiate LPS-, dopamine-, or IFN-γ-induced proinflammatory response in cultured glia cells [8–10], which can further lead to neuronal damage. Our in vivo experiments also showed that DJ-1 deficiency elevated the inflammatory tone, which sensitized microglia cells (S1 Fig) and affected the survival of dopaminergic neuron (Fig 3) by releasing more IFN-γ 5 days after LPS challenge (Fig 5). Therefore, we can further infer that microglial activation in DJ-1 KO mice accompanied with the slight increase of IFN-γ and I-TAC (S2 Fig) is prior to neuronal loss 1 day after LPS injection (Fig 3). On the other hand, it has been reported that the oxidation of dopamine produces toxic products that cause dopaminergic cell death after activation of microglia cells, and microglia with DJ-1 deficiency is sensitized to proinflammatory stimulation of dopamine, showing up-regulation of IL-1β [8, 9]. In this study, we found that the levels of IFN-γ, an upstream cytokine of IL-1β, increased in DJ-1 KO mice accompanied with an up-regulation of IL-1β levels. Therefore, dopamine stimulation may result in inflammatory profiles similar to LPS treatment, and the effects of dopamine stimulation in DJ-1 KO mice need further investigation.

DJ-1 deficiency has been demonstrated to enhance inflammation through sensitizing glia cells via different signaling pathways to directly or indirectly affect the expression of other cytokines. For example, DJ-1 knockout is known to potentiate proinflammatory response in microglia cells through JAK-STAT inflammatory signaling pathways, and to enhance proinflammatory reaction in astrocytes via p38 MAPK signaling pathway [8, 10]. Here, we demonstrated that DJ-1 deficiency can sensitize microglia cells to LPS challenge through NF-κB signaling pathway (Fig 7), and the activated microglia released more IFN-γ and its downstream mediators, such as I-TAC. Bioinformatics analysis further revealed that IFN-γ from microglia can serve as a hub protein to regulate the expression/release of other cytokines and chemokines (Fig 2). To our knowledge, we are the first to report that I-TAC expression is regulated by DJ-1.

Development of PD is highly associated with brain inflammation, as suggested by the evidence that regular administration of aspirin or other anti-inflammatory drugs can decrease the risk of PD [39]. Since DJ-1 is involved in microglial inflammatory responses, DJ-1 deficiency could facilitate the development of PD through increasing brain inflammation. Brain inflammation can be caused by either PAMPs (pathogen-associated molecular pattern molecules) from environmental microbes or DAMPs (danger-associated molecular pattern molecules) from inside of the brain. The lack of any effect of LPS, which is a model PAMP, on DJ-1 KO mice in a previous study [12] might probably be due to the intraperitoneal injection of LPS, which does not penetrate the blood-brain barrier (BBB) to reach the brain [13]. In contrast, the present study demonstrated that the local injection of LPS to the substantia nigra, which bypassed the BBB, caused more dopaminergic neuronal death in DJ-1 KO mice, suggesting that local brain inflammation, rather than a systemic inflammation, plays an important role in DJ-1 associated PD.

Activation of glial cells is a key step leading to neuroinflammation, and a hallmark of PD [40]. Previous [41] and the present studies (Figs 8, 9 and 10) demonstrated that the inflammatory factors-induced death of dopaminergic neurons occurs in neuron-glia co-cultures, but not in neuron-enriched cultures, indicating that the PD-associated neuronal damage depends on the presence of glia cells. In general, the classically cytotoxic and inflammatory mediators such as TNF-α as well as reactive oxygen species (ROS) can induce cell apoptosis of neuronal precursors, and suppress neurogenesis [42], and microglia-derived nitric oxide (NO), which is produced by inducible nitric oxide synthase (iNOS), increases the release of glutamate to cause neuronal death [43]. However, unlike IFN-γ, the mechanism of I-TAC neurotoxicity is still unclear. According to previous reports, IFN-γ, derived from activated microglia cells, can activate the expression of microglial iNOS through JAK/STAT pathway and increase the production of death-associated molecules, Fas and Fas ligand, to contribute to the loss of dopaminergic neuron cells [44–46]. It has also been demonstrated that IFN-γ can induce nigrostriatal degeneration in vivo and excessive inflammatory response is evoked in DJ-1 deficient condition through IFN-γ signaling [10, 47]. Therefore, we infer that I-TAC, which is secreted from activated microglia cells, can activate the same neurotoxic signaling pathway as IFN-γ. Since I-TAC is known as a downstream mediator of IFN-γ [48] and I-TAC is co-upregulated with IFN-γ in microglia in the DJ-1 deficient conditions (Fig 7), it can be predicted that the microglia-derived IFN-γ may act in an autocrine manner to stimulate microglia to release I-TAC. Indeed, this inference has been validated by previous literatures demonstrating that IFN-γ is an autocrine stimulatory factor of microglia and IFN-γ can induce a much higher expression of I-TAC in microglia than in other cells [48]. On the other hand, IFN-γ can also serve in a paracrine manner to act on nearby astrocytes or other inflammatory cells to evoke a cytokine storm. For example, I-TAC can be released from IFN-γ-stimulated astrocytes to act as a proinflammatory mediator to recruit IL-2-activated T cells [49–51]. Taken together, it can be inferred that DJ-1 might magnify the inflammation-induced damage through both autocrine and paracrine effects, which activate downstream mediators of inflammation.

PD is also characterized by T lymphocytes infiltration except activated microglia [51] and I-TAC can be a chemoattractant of T cell [49]. The infiltration of T lymphocytes is reported to participate in the neuroinflammation in the brains of PD patients and there is higher density of helper T lymphocytes (CD4^+^) in the brains of PD patients compared to healthy individuals [11, 52]. In addition, CD4^+^ T cell has subpopulation such as Th1 and Th17 and CD4^+^ Th1 cells are reported to secrete IFN-γ, which subsequently activates M1 microglia and enhances dopaminergic neuronal death [51]. Notably, it has been found that Th1 cells from DJ-1 KO mice increase the production of IFN-γ compared with WT mice [53]. Therefore, it is possible that T lymphocytes infiltrated into the brain in DJ-1 KO mice. However, it needs further examination to explore whether infiltration of DJ-1-deficiency T lymphocytes might mediate neuronal apoptosis after administration of LPS.

**Fig 11:**
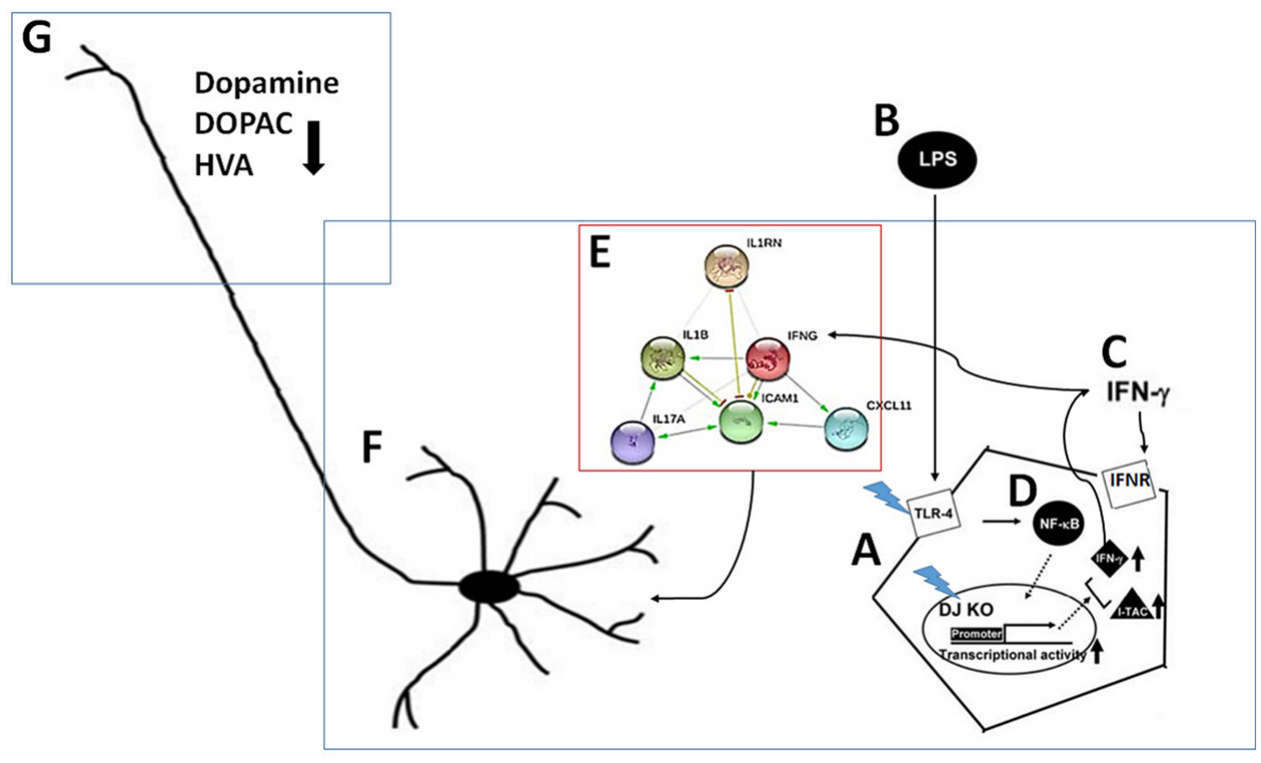
Schematic diagram of molecular and cellular mechanisms involved in DJ-1 deficiency-associated loss of dopaminergic neurons. (A) DJ-1 deficiency and (B) PAMPs may have a synergistic effect to up-regulate (C) IFN-γ and I-TAC through the activation of (D) NF-κB. The released IFN-γ then serves as a paracrine and/or an autocrine to impact on the (E) cytokine network, which in turn amplifies the inflammatory activity of microglia to cause the (F) death of dopaminergic neurons in substantia nigra and (G) depletion of striatal dopamine and its metabolites.

In summary (Fig 11), the present study demonstrated that DJ-1 deficiency and LPS challenge exert a synergistic effect to activate microglia cells, which then induce a cytokine storm via IFN-γ. The cytokine circuit amplifies the inflammatory damage to dopaminergic neurons. These results provide the mechanistic role of DJ-1 in PD. Since blockade of cytokine IFN-γ can ameliorate the microglia-mediated neuronal damage, IFN-γ may be a potential target for treating PD.

## Supporting Information

S1 Fig Microglia activation in WT and DJ-1 KO mice 1 day after LPS injection. (TIF)

S2 Fig Up-regulation of IFN-γ and I-TAC is slightly enhanced in DJ-1 knockout mice 1 day after LPS injection. (TIF)

S3 Fig The statistical results of Fig 7B. (TIF)

S4 Fig No synergistic effect of IFN-γ and I-TAC in midbrain neuron-glia mixed cultures. (TIF)
