# Supplementary figures and images for: Microglia-Derived Cytokines/Chemokines Are Involved in the Enhancement of LPS-Induced Loss of Nigrostriatal Dopaminergic Neurons in DJ-1 Knockout Mice
Source: PLoS One. 2016 Mar 16;11(3):e0151569. doi: 10.1371/journal.pone.0151569 (PMC4794203; doi:10.1371/journal.pone.0151569)

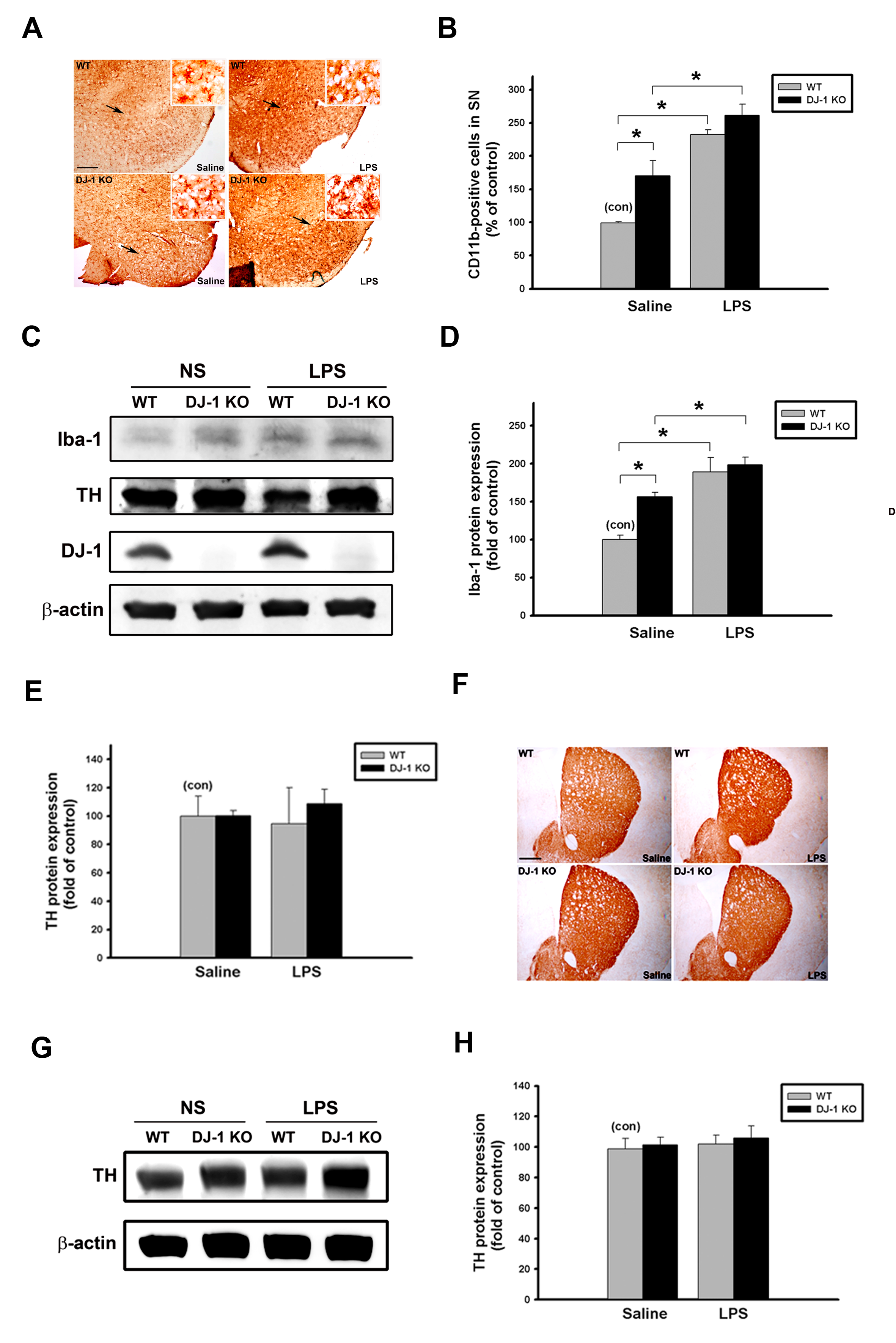

Supplement: S1 Fig — (TIF) [file pone.0151569.s001.tif]

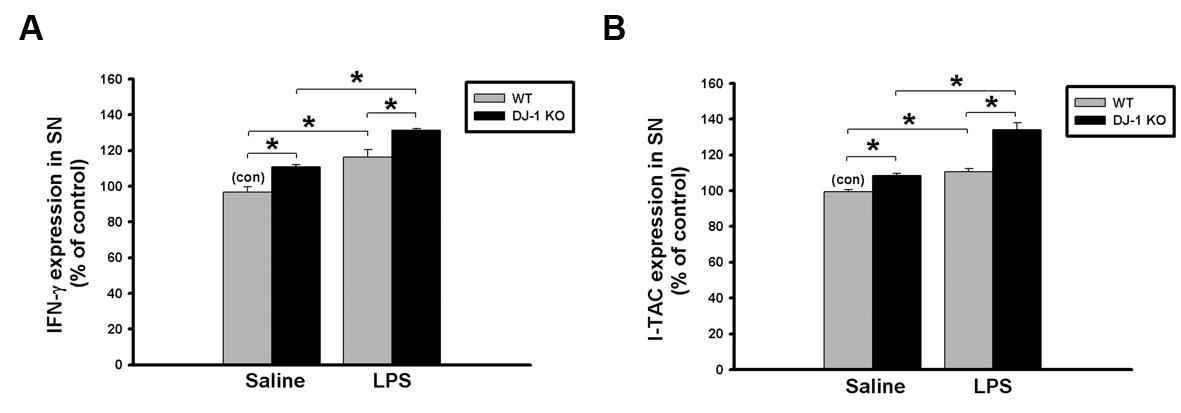

Supplement: S2 Fig — (TIF) [file pone.0151569.s002.tif]

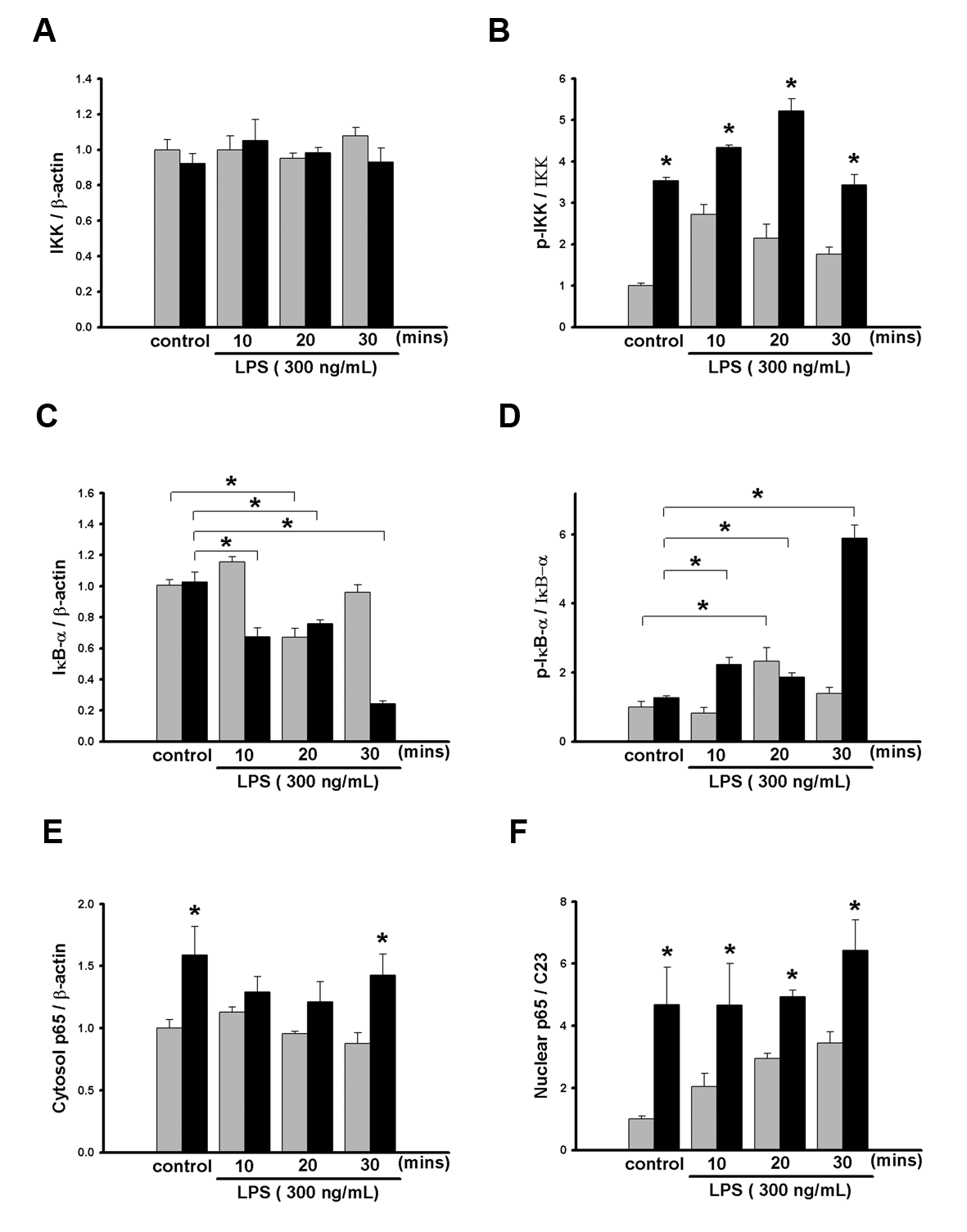

Supplement: S3 Fig — (TIF) [file pone.0151569.s003.tif]

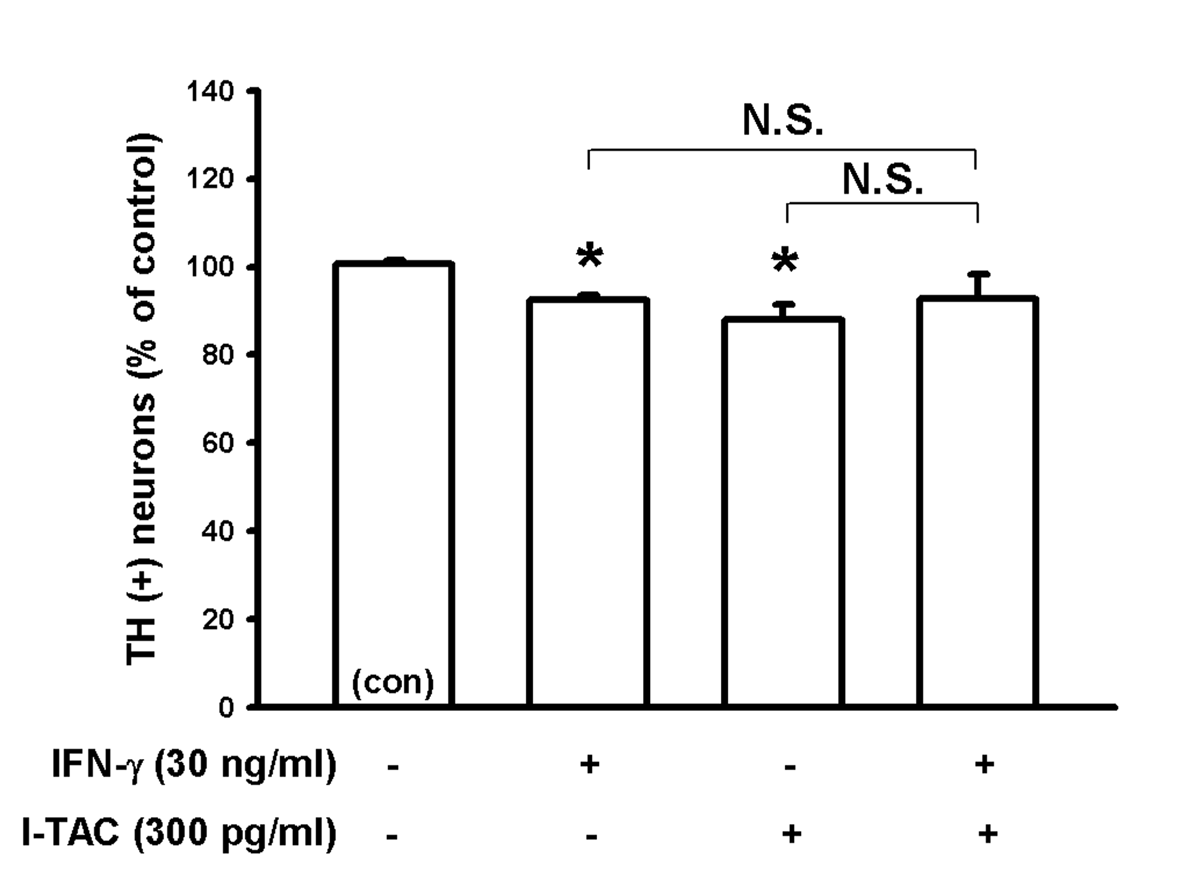

Supplement: S4 Fig — (TIF) [file pone.0151569.s004.tif]
